# Alteration of epidermal lipid composition as a result of deficiency in the magnesium transporter *Nipal4*

**DOI:** 10.1016/j.jlr.2024.100550

**Published:** 2024-04-29

**Authors:** Marino Yamaji, Yusuke Ohno, Madoka Shimada, Akio Kihara

**Affiliations:** Laboratory of Biochemistry, Faculty of Pharmaceutical Sciences, Hokkaido University, Sapporo, Japan

**Keywords:** acylceramides, ceramides, lipidomics, lipids, magnesium ion, skin, sphingolipids

## Abstract

Lipids in the stratum corneum play an important role in the formation of the skin permeability barrier. The causative gene for congenital ichthyosis, *NIPAL4*, encodes a Mg^2+^ transporter and is involved in increases in intracellular Mg^2+^ concentrations that depend on keratinocyte differentiation. However, the role of this increased Mg^2+^ concentration in skin barrier formation and its effect on the lipid composition of the stratum corneum has remained largely unknown. Therefore, in the present study, we performed a detailed analysis of epidermal lipids in *Nipal4* KO mice via TLC and MS. Compared with WT mice, the *Nipal4* KO mice showed compositional changes in many ceramide classes (including decreases in ω-*O*-acylceramides and increases in ω-hydroxy ceramides), together with increases in ω-hydroxy glucosylceramides, triglycerides, and free fatty acids and decreases in ω-*O*-acyl hydroxy fatty acids containing a linoleic acid. We also found increases in unusual ω-*O*-acylceramides containing oleic acid or palmitic acid in the KO mice. However, there was little change in levels of cholesterol or protein-bound ceramides. The TLC analysis showed that some unidentified lipids were increased, and the MS analysis showed that these were special ceramides called 1-*O*-acylceramides. These results suggest that elevated Mg^2+^ concentrations in differentiated keratinocytes affect the production of various lipids, resulting in the lipid composition necessary for skin barrier formation.

During their evolution, terrestrial organisms acquired a skin permeability barrier (skin barrier) that prevents water loss from the body, enabling them to survive on land. The skin barrier also prevents the passage of pathogens and foreign substances from the outside into the body, and a decrease in its functioning causes or increases the risk of infectious diseases, atopic dermatitis, and ichthyosis—a skin disease characterized by dry, thickened, and scaly skin (1, 2, 3). The outermost layer of the epidermis, the stratum corneum (SC), consists of corneocytes and multilayered lipid structures (lipid lamellae) (4). Two lipid structures in the SC, the lipid lamellae and the corneocyte lipid envelope, which covers the surface of corneocytes, play an important role in skin barrier formation (4, 5, 6, 7). Lipid lamellae are composed of free (nonprotein-bound) ceramides, cholesterol, and free fatty acids (FFAs), whereas corneocyte lipid envelope is composed of protein-bound ceramides (4, 6, 7).

Ceramides consist of a long-chain amino alcohol (long-chain base) and a fatty acid (FA). Mammalian ceramides contain one of five long-chain bases (dihydrosphingosine [DS], sphingosine [S], phytosphingosine [P], 6-hydroxy [OH] sphingosine [H], and 4,14-sphingadiene [SD]) and one of six FAs (non-OH FA [N], α-OH FA [A], β-OH FA [B], ω-OH FA [O], esterified ω-OH FA [EO], and protein-bound FA [PB-]), and each ceramide class is represented by a combination of the abbreviations of the long-chain base and the FA (Supplemental Fig. S1). A variety of ceramides with different combinations of long-chain base and FA exist in the SC (8, 9). The ω-*O*-acylceramides (EO-type ceramides) are unique among ceramides in that the FA portion is ω-hydroxylated and esterified with a linoleic acid (C18:2 FA), and they are important for the formation and maintenance of lipid lamellae (3, 7, 8, 10, 11). PB-type ceramides are derived from ω-*O*-acylceramides (6, 7). In their synthesis, the linoleic acid moiety of ω-*O*-acylceramides is modified to epoxy-enone, followed by covalent binding to cysteine residues of the surface proteins of corneocytes (12, 13). Of all the ceramides, ω-*O*-acylceramides and protein-bound ceramides are especially important for skin barrier formation. In fact, mutations in the genes involved in the production of either of these groups cause congenital ichthyosis in humans (14, 15, 16, 17, 18, 19, 20), and KO mice of these genes are neonatal lethal due to skin barrier defects (10, 11, 12, 21, 22, 23, 24, 25, 26, 27).

*NIPAL4* has been identified as the causative gene of autosomal recessive congenital ichthyosis (17), and 34 autosomal recessive congenital ichthyosis-causing mutations have been reported to date (28). NIPAL4 belongs to the NIPA (nonimprinted in Prader-Willi/Angelman syndrome) family, which also includes NIPA1, NIPA2, and NIPAL1 (also known as NIPA3) (29, 30, 31). Analyses using the *Xenopus laevis* oocyte expression system have shown that they all exhibit Mg^2+^ inward transport activity (32). Previously, we generated *Nipal4* KO mice and revealed that they exhibit neonatal lethality (due to skin barrier abnormalities), hyperkeratosis, and impaired lipid lamella formation (33). We also found that Mg^2+^ concentration was increased in a differentiation-dependent manner in keratinocytes prepared from WT mice, whereas this increase in *Nipal4* KO mouse keratinocytes was about half that in WT mouse keratinocytes. In addition, ceramide class composition was altered in *Nipal4* KO mouse epidermis, including a decrease in the quantity of ω-*O*-acylceramides to about half that of WT mouse epidermis. However, the ceramide classes measured in that report were limited, and lipids other than ceramides were not measured. Furthermore, the molecular mechanism of how the impaired Mg^2+^ import by NIPAL4 causes changes in ceramide composition or skin barrier abnormalities remained unclear.

Recently, we established an LC/MS/MS system that can measure ceramides quantitatively with high sensitivity, and this revealed that 26 classes of ceramides exist in the human SC and 21 classes in the mouse SC (9, 34). In the present study, we carried out a comprehensive measurement of ceramides, using the established LC/MS/MS system, as well as of other lipids. We found that the quantities of many epidermal lipids are altered in *Nipal4* KO mice. These findings reveal the physiological significance of NIPAL4 in skin barrier formation: increased Mg^2+^ concentration in differentiated keratinocytes induces changes in the composition of the lipids required for skin barrier formation.

## Materials and Methods

### Mice

The *Nipal4* KO, *Cyp4f39* KO, *Elovl1* KO, and *Fatp4* KO mice, all on a C57BL/6J background, used in this study were generated previously (10, 11, 19, 25, 33). WT C57BL/6J mice for backcrossing were purchased from Sankyo Lab Service (Tokyo, Japan). To prepare the homozygous *Nipal4* KO mice, male and female heterozygous KO mice were crossed, and littermate WT and homozygous KO mice were subjected to analyses. Mice were maintained in a specific pathogen-free environment with a room temperature of 23 ± 1°C, humidity of 50 ± 5%, 12 h light/dark cycle, and free access to a standard chow diet (PicoLab Rodent Diet 20; LabDiet, St. Louis, MO) and water. Animal experiments were approved by the institutional animal care and use committee of Hokkaido University.

### Lipid extraction

Dorsal skin dissected from postnatal day 0 mice was incubated with 5 mg/ml dispase (Thermo Fisher Scientific, Waltham, MA) in PBS for 16 h at 4°C, and then separated into epidermis and dermis. Epidermis (∼1.5 mg) was transferred to a tube containing zirconia beads and suspended in 450 μl chloroform/methanol (1:2, v/v). For LC/MS/MS analysis, the following internal standards were added to the samples: deuterium-labeled ceramide standards (50 pmol *N*-palmitoyl[*d*_9_] D-*erythro*-sphingosine [*d*_9_-C16:0 NS], 50 pmol *N*-(2′-(*R*)-hydroxypalmitoyl[*d*_9_]) D-*erythro*-sphingosine [*d*_9_-C16:0 AS], 100 pmol *N*-palmitoyl[*d*_9_] dihydrosphingosine [*d*_9_-C16:0 NDS], 100 pmol *N*-(2′-(*R*)-hydroxypalmitoyl[*d*_9_]) D-*erythro*-dihydrosphingosine [*d*_9_-C16:0 ADS], 12.5 pmol *N*-palmitoyl[*d*_9_] D-*ribo*-phytosphingosine [*d*_9_-C16:0 NP], 12.5 pmol *N*-palmitoyl[*d*_9_] 6-(*R*)-hydroxysphingosine [*d*_9_-C16:0 NH], 12.5 pmol *N*-(2′-(*R*)-hydroxypalmitoyl[*d*_9_]) D-*ribo*-phytosphingosine [*d*_9_-C16:0 AP], 12.5 pmol *N*-(2′-(*R*)-hydroxypalmitoyl[*d*_9_]) 6-(*R*)-hydroxysphingosine [*d*_9_-C16:0 AH], all from Avanti Polar Lipids, Alabaster, AL), 3.97 pmol *d*_5_-triglyceride (TG) (*d*_5_-TG ISTD Mix I; Avanti Polar Lipids), and 250 pmol *d*_31_-palmitic acid (Cayman Chemical, Ann Arbor, MI). Epidermis was crushed (4,500 rpm, 4°C, 1 min) using a Micro Smash MS-100 (Tommy Seiko, Tokyo, Japan). After centrifugation (20,400 *g*, room temperature, 3 min), the supernatant was collected. After 450 μl of chloroform/methanol (1:2, v/v) was added to the pellet, the epidermis was crushed again, centrifuged, and the supernatant collected. The resulting pellets were used for extraction of protein-bound ceramides (see below). The two supernatants were combined, mixed with 300 μl of chloroform, and 540 μl of H_2_O, and separated into two phases by centrifugation (20,400 *g*, room temperature, 3 min). The lower layer (organic phase) was collected and dried. The lipids obtained were suspended in 750 μl of chloroform/methanol (1:2, v/v), diluted eightfold with chloroform/methanol (1:2, v/v), and 5 μl of each was subjected to LC/MS/MS analysis as described below.

Protein-bound ceramides were extracted from the epidermal pellets as follows. To remove free lipids, 1 ml of methanol was added to the pellets and mixed vigorously. After centrifugation (2,600 *g*, room temperature, 3 min), the supernatant was removed. The same procedure was repeated twice. Next, 1 ml of 95% methanol was added to the pellet, mixed vigorously, and incubated at 60°C for 2 h. After centrifugation (2,600 *g*, room temperature, 3 min), the supernatant was removed. The same procedure was performed again. The resulting pellets were suspended in 1 ml of 1 M KOH in 95% methanol containing internal standards (50 pmol *d*_9_-C16:0 AS, 200 pmol *d*_9_-C16:0 ADS, 5 pmol *d*_9_-C16:0 AH, and 5 pmol *d*_9_-C16:0 AP) and incubated at 60°C for 2 h to release ω-OH ceramides (O-type ceramides) by cleaving ester bonds. After neutralization with 1 ml of 1 M acetic acid, samples were mixed vigorously with 1 ml of chloroform for 1 min and centrifugated (2,600 *g*, room temperature, 3 min). The lower layer (organic phase) was collected, dried, suspended in 100 μl of chloroform/methanol (1:2, v/v), and diluted 100-fold with chloroform/methanol (1:2, v/v), and 5 μl of each was subjected to LC/MS/MS analysis for ω-OH ceramide measurement.

The FAs were converted to *N*-(4-aminomethylphenyl) pyridinium (AMP)-amide FAs via reaction with AMP using the AMP^+^ MaxSpec Kit (Cayman Chemical) to increase the ionization efficiency of the FAs for LC/MS/MS analysis. Lipid samples (10 μg of epidermis) were subjected to the AMP-amide FA derivatization, according to the manufacturer’s protocols, and diluted to 100 μl with methanol, of which 5 μl was used for LC/MS/MS analysis as described below.

### Lipid analyses by TLC

Lipids (1 mg of epidermis) were spotted onto normal-phase TLC plates (Silica Gel 60 TLC plates; Merck Millipore, Darmstadt, Germany) and separated via one of the following development systems: A, suitable for separating ceramides and glucosylceramides (Glc-ceramides), or B, suitable for separating lipids with higher hydrophobicity than ceramides. Development system A: *i)* chloroform/methanol/water (40:10:1, v/v/v), developed to 2 cm from the bottom of the TLC plate, dried, and then developed to 5 cm from the bottom again; *ii)* chloroform/methanol/acetic acid (94:4:1, v/v/v), developed to the top; and *iii)* hexane/diethyl ether/acetic acid (65:35:1, v/v/v), developed to the top, dried, and then developed to the top again. Development system B: hexane/diethyl ether/acetic acid (65:35:1, v/v/v), developed to the top. Lipids were stained with copper phosphate reagent (3% [w/v] copper acetate in 8% [v/v] phosphoric acid) at 180°C for 3 min.

### Identification of 1-*O*-acylceramides

Lipids extracted from epidermis were separated using normal-phase TLC plates (Silica Gel 60 TLC plates; Merck) with hexane/diethyl ether/acetic acid (65:35:1, v/v/v). Silica gel containing unidentified lipids (which later turned out to be 1-*O*-acylceramides) was scraped from the TLC and incubated with 100 μl of chloroform/methanol (1:2, v/v) for 1 h at 37°C. After centrifugation (20,400 *g*, room temperature, 3 min), the eluted lipids were recovered. The elution procedure was repeated, and the second supernatant was combined with the first. The samples were dried, suspended in 100 μl hexane, and mixed with methanol/water (9:1, v/v). After centrifugation (20,400 *g*, room temperature, 3 min), the upper layer was recovered. The lower layer was mixed with 100 μl hexane and centrifuged again. This second upper layer was collected and combined with the first. The samples were dried, suspended in 100 μl chloroform/methanol (1:2, v/v), and subjected to LC/MS analysis as described below.

### Lipid analyses via LC/MS and LC/MS/MS

Scan analyses via LC/MS were performed using an LC-coupled quadrupole-time-of-flight mass spectrometer (Xevo G2 QTof; Waters Corp, Milford, MA). Product-ion scanning and multiple reaction monitoring (MRM) analyses via LC/MS/MS were conducted using an LC-coupled triple quadrupole mass spectrometer (Xevo TQ-S; Waters). For the LC separation, a reversed-phase column (AQUITY UPLC CSH C18 column; particle size, 1.7 μm; inner diameter, 2.1 mm; 100 mm long; Waters Corp) and the binary gradient solvent system were used as described previously (13). The column temperature was set at 55°C. Electrospray ionization was performed using the following parameters: capillary voltage, 2.5 kV; sampling cone, 30 V; source offset, 50 V; desolvation temperature, 650°C; desolvation gas flow, 1,200 L/h; cone gas flow, 150 L/h. The scan analyses by LC/MS were performed for *m/z* = 300–1,000. In the product-ion scanning via LC/MS/MS, precursor ions with *m/z* = 916.9 were fragmented at a collision energy of 45 eV, and the product ions were scanned in the range *m/z* = 200–1,000. Measurement of each ceramide class in the MRM mode of LC/MS/MS was performed as described previously (9, 35). The *m/z* values of the precursor (Q1) and product (Q3) ions and the collision energies used in the MRM analysis are listed in Supplemental Tables S1–S6. The quantities of ceramides, TGs, and FFAs were calculated from the ratio of the peak area of each lipid species to that of the deuterium-labeled internal standard corresponding to each lipid class. We quantified O- and EO-type ceramides using the deuterium-labeled A-type ceramide standards, since deuterium-labeled O-/EO-type ceramides are not commercially available. Similarly, we quantified Glc-ceramides using the corresponding deuterium-labeled ceramide standards. The MassLynx (https://www.waters.com/nextgen/en/products/informatics-and-software/mass-spectrometry-software/masslynx-mass-spectrometry-software.html) software (Waters) was used for data analysis.

### RNA sequencing

Mice at embryonic day 18.5 were collected via Caesarean section, and the dorsal skin was dissected. The skin, suspended in PBS, was heated at 55°C for 3 min, and the epidermis and dermis were separated. Total RNAs were extracted from the epidermis using TRIzol (Thermo Fisher Scientific), and sequencing libraries were prepared using the NEBNext Poly(A) mRNA Magnetic Isolation Module (New England Biolabs, Ipswich, MA) and NEBNext Ultra II Directional RNA Library Prep Kit (New England Biolabs) according to the manufacturer’s protocol. The library was then sequenced on the Illumina NovaSeq 6000 (Illumina, San Diego, CA), and 150 bp paired-end reads (6 Gb) were generated. The quality checking, trimming, and mapping of the reads were performed using FastQC (https://www.bioinformatics.babraham.ac.uk/projects/fastqc/, version 0.11.7) (36), Trimmomatic (http://www.usadellab.org/cms/?page=trimmomatic, version 0.38) (37), and HISAT2 (http://daehwankimlab.github.io/hisat2/, version 2.1.9) (38), respectively. The mapped reads were counted using featureCounts (https://subread.sourceforge.net/featureCounts.html, version 1.6.3) (39, 40). The read counts were normalized to transcripts per million.

### In vitro transacylase assay

We performed an in vitro transacylase assay using PNPLA1 translated by a cell-free translation system and ω-OH ceramide and linoleic acid-containing TG as substrates as described previously (41).

### Statistical analyses

Data are presented as means + standard deviation. After conducting a test for normality (Shapiro–Wilk test) in JMP13 (https://www.jmp.com/en_us/home.html, SAS Institute, Cary, NC), we evaluated the significance of differences by conducting Welch’s *t* test in Microsoft Excel (https://www.microsoft.com/en-us/microsoft-365/excel, Microsoft, Redmond, WA) or Dunnett’s test (for multiple comparisons) in JMP13. *P*-values of < 0.05 were considered significant.

## Results

### Changes in epidermal lipid composition in *Nipal4* KO mice

**Fig. 1:**
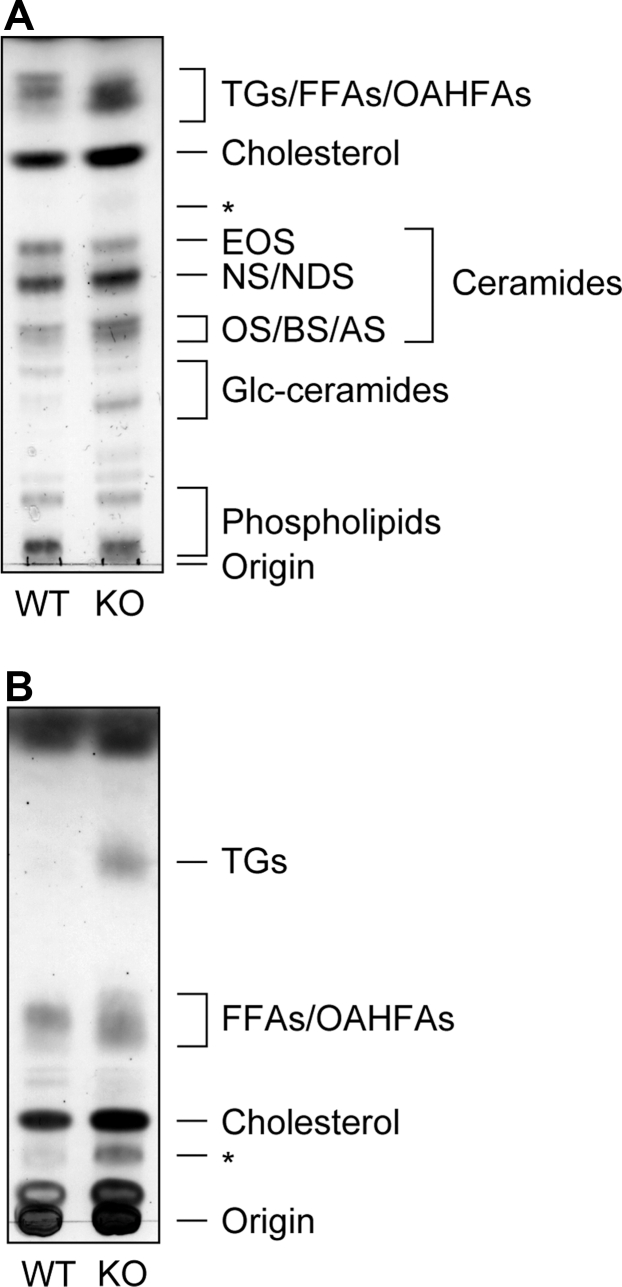
Lipid composition changes in the epidermis of *Nipal4* KO mice. Lipids were extracted from the epidermis of postnatal day 0 WT and *Nipal4* KO mice. Lipids (1 mg of epidermis) were separated via normal-phase TLC using two different solvent systems (A, suitable for the separation of ceramides and Glc-ceramides; B: suitable for separation of lipids more hydrophobic than ceramides) and stained using copper phosphate reagent. Asterisks indicate an unidentified lipid.

To reveal changes in the lipid composition of the epidermis of *Nipal4* KO mice, lipids extracted from the epidermis of postnatal day 0 WT and *Nipal4* KO mice were separated via TLC using two different solvent systems and detected via copper phosphate staining. We found the following changes in epidermal lipids in *Nipal4* KO mice compared to WT mice: altered ceramide composition (a decrease in ω-*O*-acylceramide EOS and increases in some ceramide classes with similar TLC mobility as NS/NDS and OS/BS/AS), increases in specific Glc-ceramide class and TGs, and changes in the patterns of bands near FFAs and *O*-acyl-ω-OH FAs (OAHFAs) (Fig. 1).

### Changes in ceramide composition in *Nipal4* KO mice

**Fig. 2:**
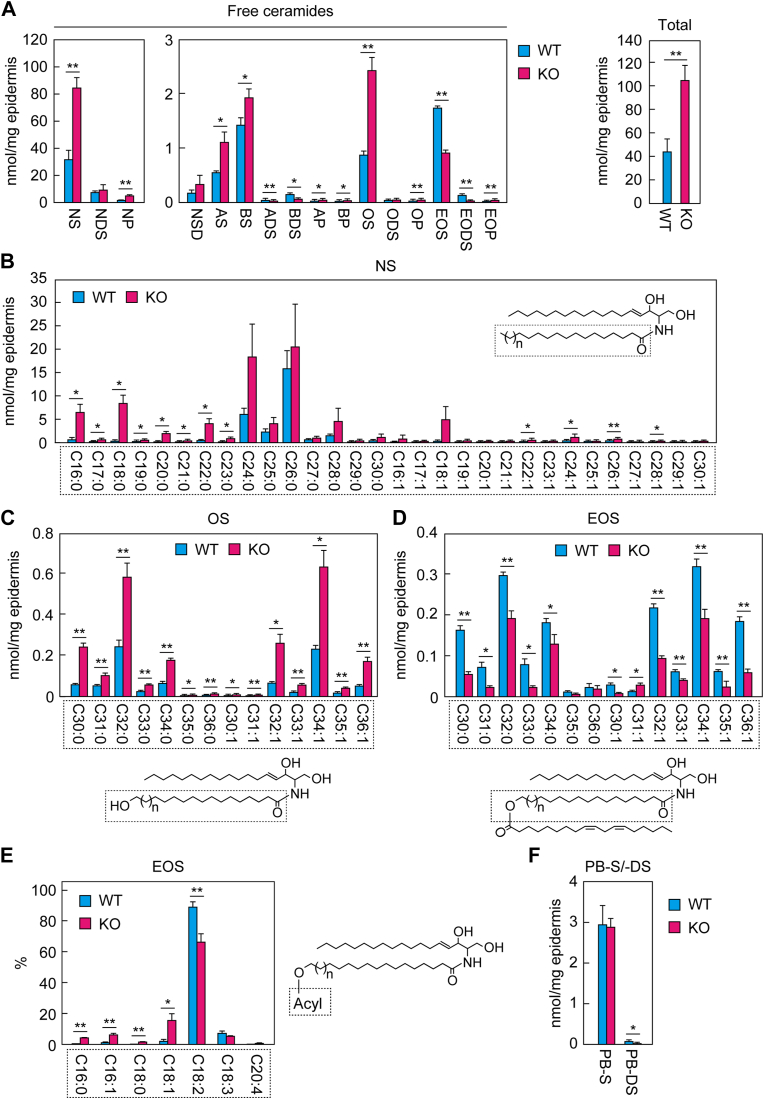
Changes in ceramide composition in the epidermis of *Nipal4* KO mice. Lipids were prepared from the epidermis of postnatal day 0 WT and *Nipal4* KO mice. Free (nonprotein-bound) ceramides (A–E) and protein-bound ceramides (F) were quantified via LC/MS/MS. The quantity of each free ceramide class and the total quantity (A), FA or ω-OH FA composition and structures of NS (B), OS (C), and EOS (D), *O*-acyl-chain composition and structure of EOS (E), and the quantity of protein-bound ceramide classes PB-S and PB-DS (F) are shown. Values presented are mean + standard deviation (n = 3, ∗*P* < 0.05, ∗∗*P* < 0.01; Welch’s *t* test). FA, fatty acid; PB, protein-bound.

We previously measured quantities of NS, EOS, AS, OS, NDS, and NP in the epidermis of *Nipal4* KO mice via LC/MS/MS analysis and reported increases in NS, OS, and NP and decreases in EOS, AS, and NDS compared to WT mice (33). However, we did not measure all ceramide classes in that study. Subsequently, we have established a more quantitative and comprehensive ceramide-measurement LC/MS/MS system and found that the following ceramide classes are present in mice: NS, NDS, NP, NSD, AS, BS, ADS, BDS, AP, BP, OS, ODS, OP, EOS, EODS, EOP, PB-S, and PB-DS (9). In the present study, we applied the newly established ceramide-measurement method to the epidermis of postnatal day 0 WT and *Nipal4* KO mice. In WT mice, NS was the most abundant free ceramide class, followed by NDS, EOS, BS, OS, AS, and NP in that order (Fig. 2A); NSD, ADS, BDS, AP, BP, ODS, OP, EODS, and EOP were minor classes. In *Nipal4* KO mice, NS, NP, AS, BS, AP, BP, OS, OP, and EOP were increased compared to the WT mice, whereas ADS, BDS, EOS, and EODS were decreased. The most prominent changes in levels were increases in NS and the ω-OH ceramide OS (both 2.5-fold those in WT) and decreases in the acylceramide EOS (about half). The total quantity of free ceramides in *Nipal4* KO mice was approximately double than in the WT mice.

Each ceramide class contains multiple species in which the FA moiety has different chain lengths and/or degrees of unsaturation (saturated or monounsaturated). In the case of NS, the FA chain length ranged from C16 to C30, and the C26:0 species was the most abundant in WT mice, followed by C24:0 (Fig. 2B). Although the quantity of the C26:0 species in *Nipal4* KO mice was comparable to that in WT mice, the quantities of many species with ≤C23 were higher.

The ω-OH FA portion of OS and EOS in the epidermis of WT mice were C30 to C36, and C32:0 and C34:1 were the most abundant species in these classes (Fig. 2C, D). In *Nipal4* KO mice, OS levels were increased 2- to 4-fold compared to WT mice, regardless of the chain length (Fig. 2C). The levels of many EOS species were lower in *Nipal4* KO mice than those in WT mice, especially those with C30:0, C31:0, C33:0, C30:1, C32:1, C35:1, and C36:1 (less than half of those in WT mice; Fig. 2D). It is known that most of the *O*-acyl chain of ω-*O*-acylceramides consists of linoleic acid (C18:2) (42). Accordingly, we found that 89% of the *O*-acyl chain of ω-*O*-acylceramides in WT mouse epidermis was linoleic acid, with palmitic acid (C16:0), palmitoleic acid (C16:1), stearic acid (C18:0), oleic acid (C18:1), and linolenic acid (C18:3) being much less abundant (Fig. 2E). Although ω-*O*-acylceramides containing linoleic acid were decreased in *Nipal4* KO mice, those containing other FAs increased. Of these, oleic acid-containing acylceramides were the most abundant and accounted for 16% of the total ω-*O*-acylceramides.

Although protein-bound ceramides are essential for skin barrier formation (5, 6, 7), we did not measure their levels in *Nipal4* KO mice in our previous study (33). Here, we measured them via LC/MS/MS and found no differences between WT and *Nipal4* KO mice in quantities of the major protein-bound ceramide class PB-S (Fig. 2F). However, that of PB-DS, a less abundant protein-bound ceramide class, was reduced in *Nipal4* KO mice. In summary, we have provided an overall picture of the changes in ceramide composition in *Nipal4* KO mice.

### Increased levels of Glc-ceramides containing OS (GlcOS) in *Nipal4* KO mice

**Fig. 3:**
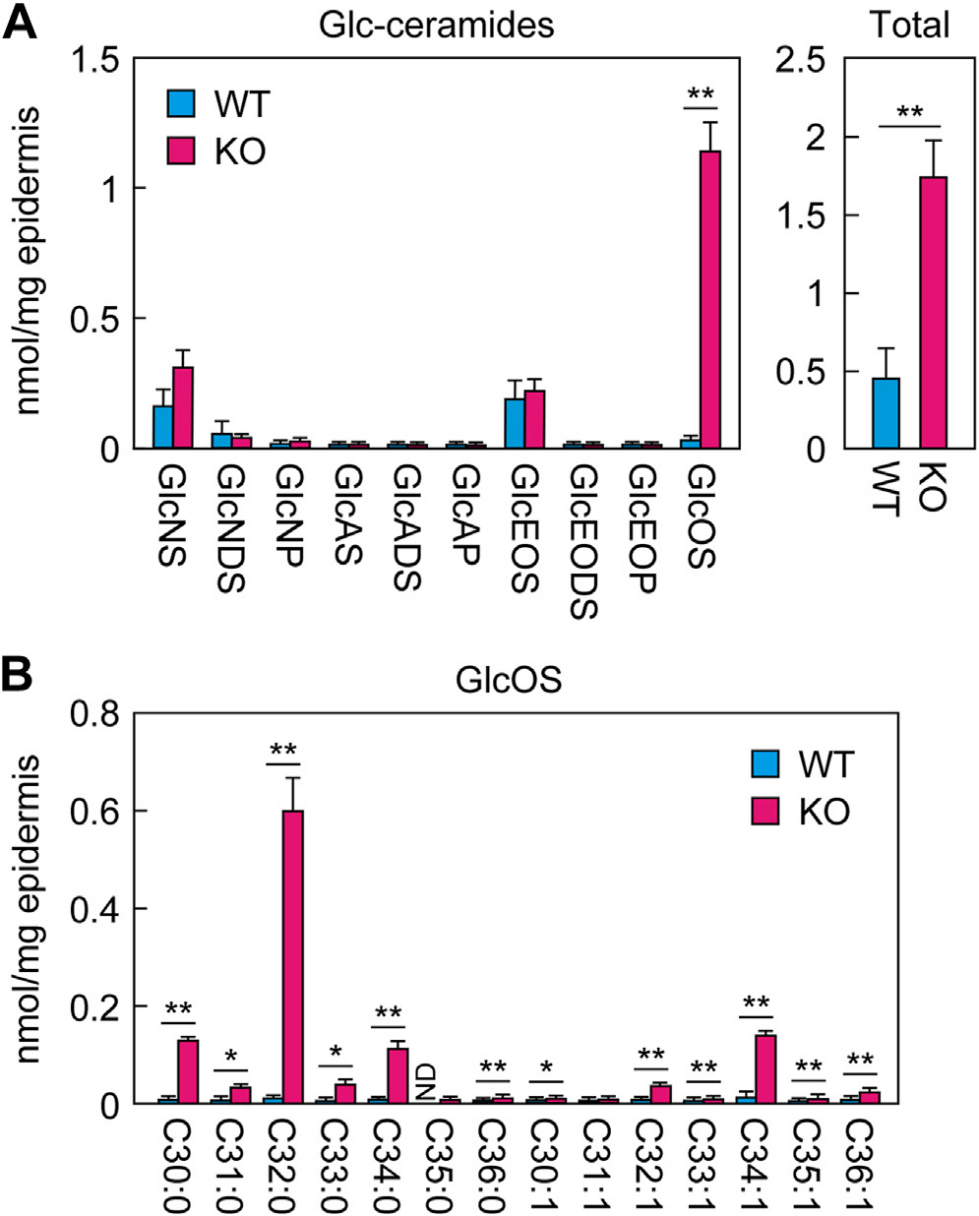
Increased ω-OH Glc-ceramide GlcOS levels in the epidermis of *Nipal4* KO mice. Lipids were extracted from the epidermis of postnatal day 0 WT and *Nipal4* KO mice, and Glc-ceramides were quantified via LC/MS/MS. The quantity of each Glc-ceramide class and the total quantity (A) and ω-OH FA composition of GlcOS (B) are shown. Values presented are mean + standard deviation (n = 3, ∗*P* < 0.05, ∗∗*P* < 0.01; Welch’s *t* test). ND, not detected.

In the TLC analysis (Fig. 1A), we found an increase in a certain class of Glc-ceramides. To reveal which Glc-ceramide class was increased, we quantified each class via LC/MS/MS. In WT mice, Glc-ceramides containing NS and EOS as the ceramide moieties (GlcNS and GlcEOS, respectively) were the most abundant classes (Fig. 3A). In *Nipal4* KO mice, there was little change in the quantities of many Glc-ceramide classes, whereas that of GlcOS was greatly increased (approximately 40-fold than in WT mice). The total quantity of Glc-ceramides was increased ∼4-fold in *Nipal4* KO mice compared to WT mice. The ω-OH FA portion of GlcOS in WT mice ranged from C30 to C36, with the most abundant being C32:0 (Fig. 3B). In *Nipal4* KO mice, all species were increased, regardless of chain length. In conclusion, GlcOS was specifically increased among the Glc-ceramide classes in *Nipal4* KO mice.

### Increased TG levels in *Nipal4* KO mice

**Fig. 4:**
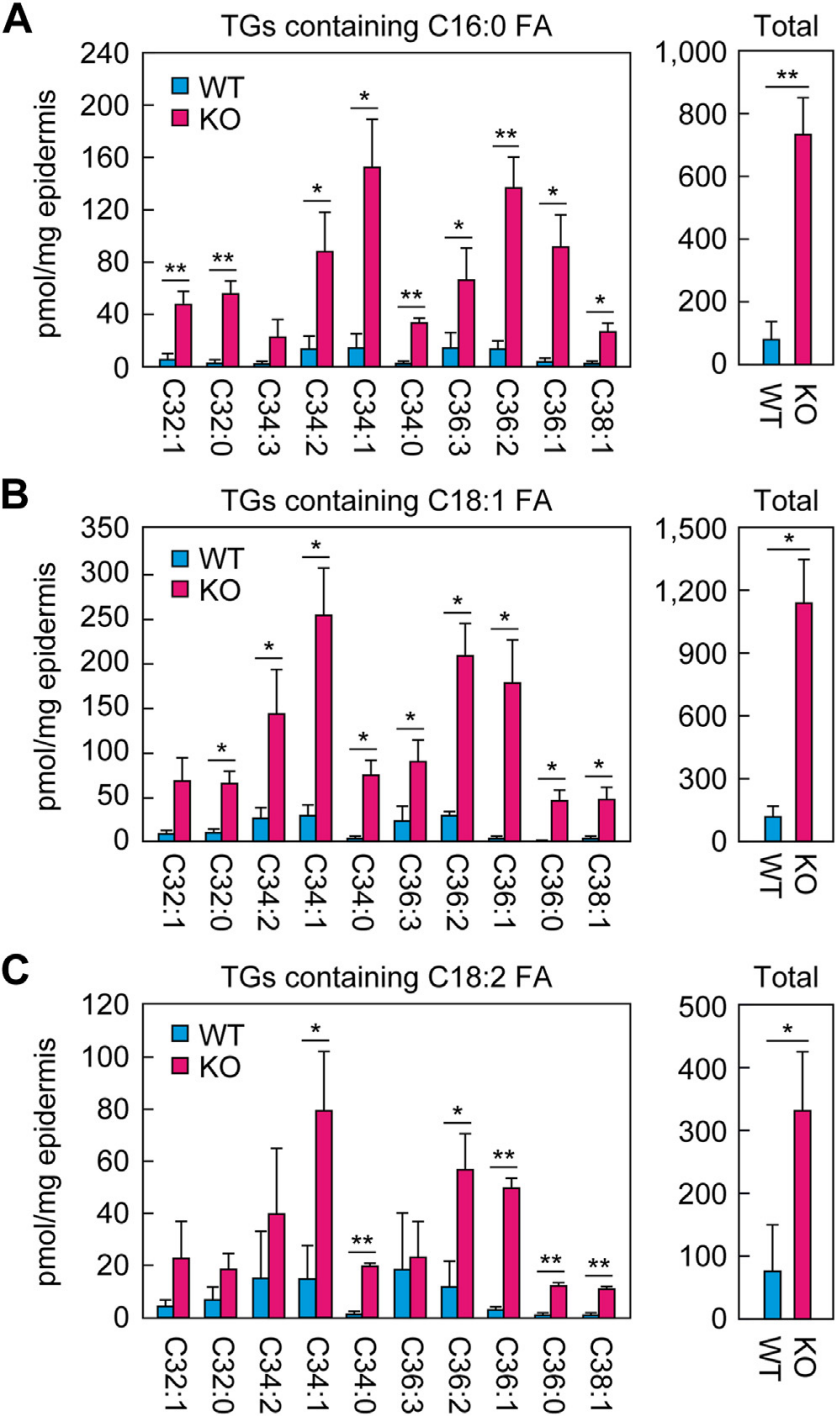
Increased TG levels in the epidermis of *Nipal4* KO mice. Lipids were extracted from the epidermis of postnatal day 0 WT and *Nipal4* KO mice, and C16:0 FA-containing TGs (A), C18:1 FA-containing TGs (B), and C18:2 FA-containing TGs (C) were quantified via LC/MS/MS. The top 10 species in terms of abundance in *Nipal4* KO mice for each TG are shown in the left panels, and their total quantities are shown in the right panels. The labels on the *x*-axes of the graphs in the left-hand panels indicate the total chain length and degree of unsaturation of the two acyl chains other than the one indicated at the top of that panel (A, C16:0; B, C18:1; C, C18:2). Values presented are mean + standard deviation (n = 3, ∗*P* < 0.05, ∗∗*P* < 0.01; Welch’s *t* test). FA, fatty acid; TG, triglyceride.

Our TLC analysis revealed that TG levels were increased in *Nipal4* KO mice (Fig. 1B). To determine which TG species were increased, we performed MRM analyses via LC/MS/MS. Since specific precursor and product ions are selected in MRM analysis, only one acyl chain of the TGs can be specified, with the remaining two acyl chains calculated as the sum of the number of carbons and the degree of unsaturation. In *Nipal4* KO mice, the quantities of TG species containing palmitic acid (C16:0) or oleic acid (C18:1) were increased compared to WT mice in all species measured, and the total quantities of both types were ∼9-fold higher in KO mice (Fig. 4A, B). Of TGs containing linoleic acid (C18:2), many species were increased in *Nipal4* KO mice compared to WT mice, with the total quantity being 4.4-fold higher (Fig. 4C). These results indicate that TG species were increased overall in *Nipal4* KO mice.

### Changes in FFA and OAHFA levels in *Nipal4* KO mice

**Fig. 5:**
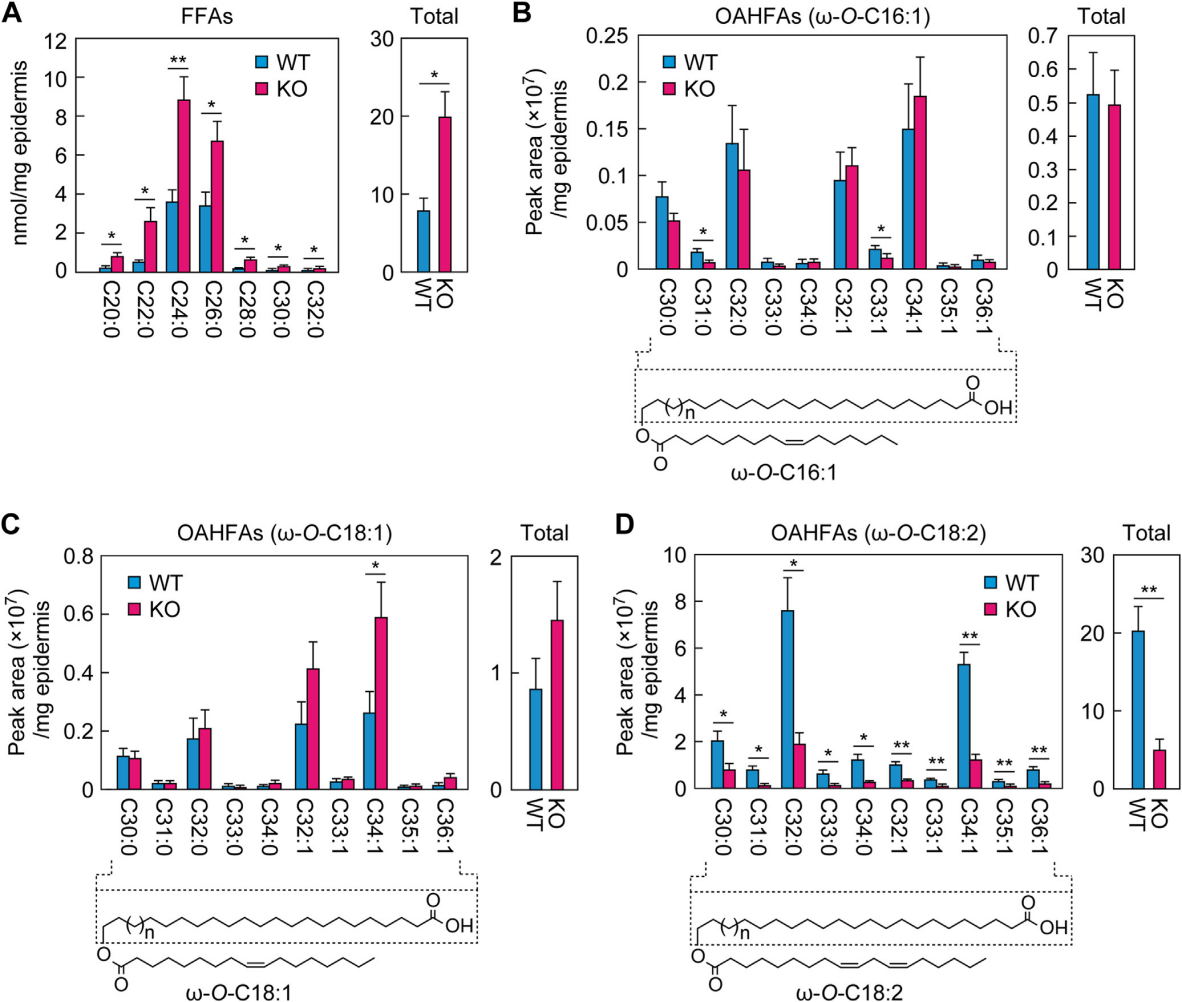
Increased FFAs and reduced C18:2 FA-containing OAHFAs in epidermis of *Nipal4* KO mice. Lipids were extracted from the epidermis of postnatal day 0 WT and *Nipal4* KO mice. After derivatization with AMP amide, FFAs (A) and OAHFAs (B–D) were quantified via LC/MS/MS analysis. The FA or ω-OH FA composition (left) of FFAs (A), C16:1 FA-containing OAHFAs (B), C18:1 FA-containing OAHFAs (C), and C18:2 FA-containing OAHFAs (D) and their total quantities (right) are shown. The structures of the OAHFAs with the analyzed ω-OH FA moiety indicated are shown below each graph (B–D). Values presented are mean + standard deviation (n = 3, ∗*P* < 0.05, ∗∗*P* < 0.01; Welch’s *t* test). AMP, *N*-(4-aminomethylphenyl) pyridinium; FFAs, free fatty acids; OAHFAs, O-acyl-omega-hydroxy fatty acids.

We observed differences in the pattern of lipid bands around FFAs/OAHFAs between WT and *Nipal4* KO mice in the TLC analysis (Fig. 1B). To investigate the cause of this difference, we first quantified FFAs via LC/MS/MS. The quantities of all saturated FFAs from C20 to C32 were increased in *Nipal4* KO mice compared to WT mice, and the total levels were 2.5-fold higher (Fig. 5A). We then quantified OAHFAs via LC/MS/MS analysis. There was little difference in the quantities of C16:1 FA-containing OAHFAs or C18:1 FA-containing OAHFAs between WT and *Nipal4* KO mice (Fig. 5B, C). However, all species of C18:2 FA-containing OAHFAs were reduced in *Nipal4* KO mice to 10%–40% of those in WT mice and to 24% in total (Fig. 5D). Of the C18:2 FA-containing OAHFA species, those with C32:0 and C34:1 ω-OH FAs were the most abundant both in WT and KO mice, and this ω-OH FA composition was similar to that of EOS (Fig. 2D). This suggests that the C18:2 FA-containing OAHFAs are produced from the acylceramide EOS via degradation (removal of the sphingosine moiety). The lower quantities of C18:2 FA-containing OAHFAs in *Nipal4* KO mice may reflect the reduced levels of EOS.

### Increased 1-*O*-acylceramides in *Nipal4* KO mice

**Fig. 6:**
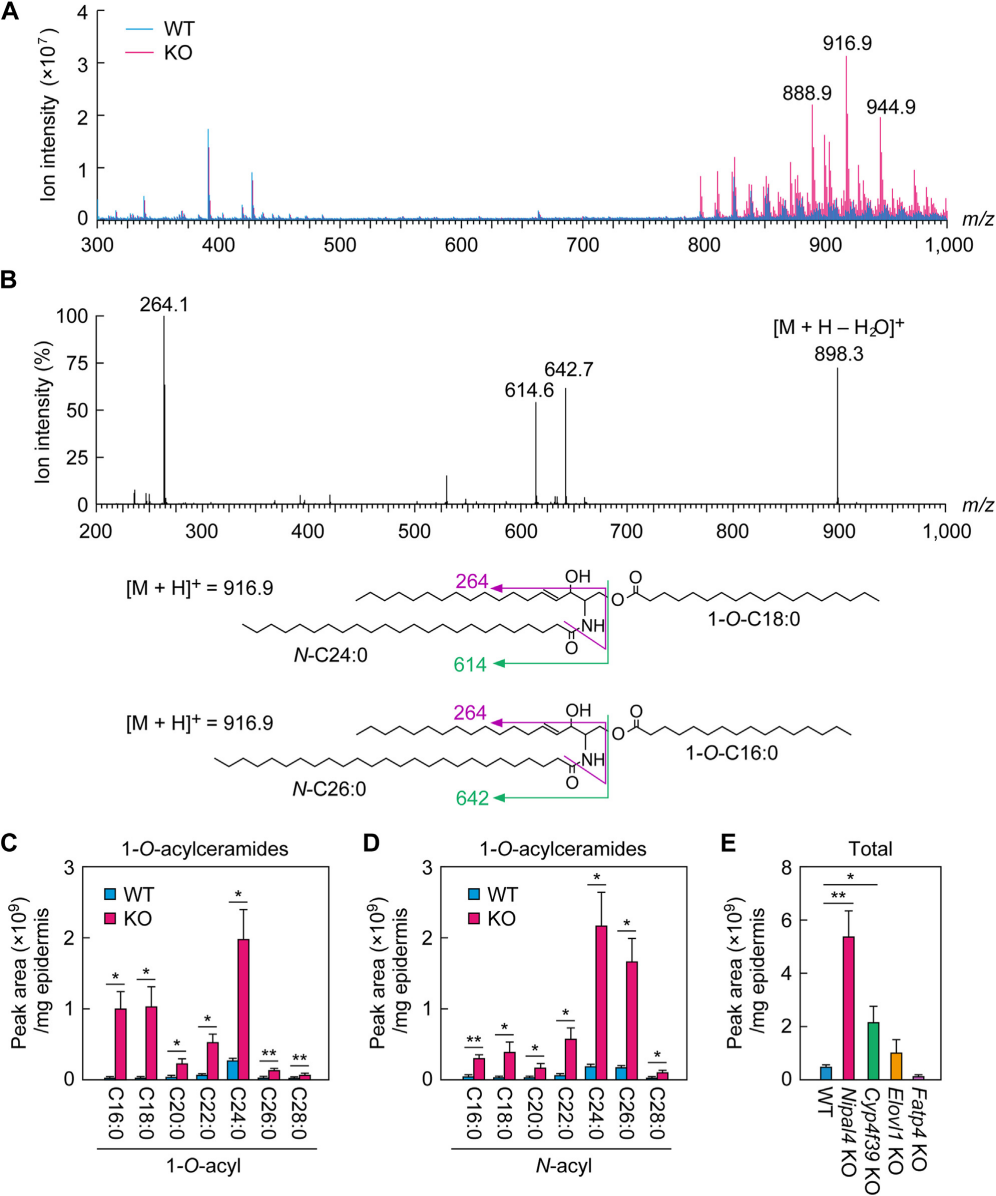
Increased 1-*O*-acylceramides in the epidermis of *Nipal4* KO mice. A–E: Lipids were extracted from the epidermis of postnatal day 0 WT (A–E), *Nipal4* KO (A–E), *Cyp4f39* KO (E), *Elovl1* KO (E), and *Fatp4* KO mice (E). A and B: After separation of lipids via TLC, an unidentified lipid was scraped from the TLC and eluted using chloroform/methanol = 1:2 (v/v). A: Full-scan LC/MS analyses were performed (*m/z* 300–1,000; positive-ion mode). The mass spectra are shown. B: Product-ion scan analysis was conducted on the molecular ion with *m/z* 916.9. The predicted structures of two 1-*O*-acylceramides with *m/z* 916.9 and their product ions are shown below. C–E: 1-*O*-acylceramides were quantified via LC/MS/MS using MRM mode. Values presented indicate the *O*-acyl (C) and *N*-acyl (D) chain composition of 1-*O*-acylceramides and the total quantities of 1-*O*-acylceramides (E). Values presented are mean + standard deviation (n = 3; ∗*P* < 0.05, ∗∗*P* < 0.01; Welch’s *t* test [C, D]; Dunnett’s test versus WT mice [E]).

In the TLC analyses, an unidentified lipid was increased in *Nipal4* KO mice compared to WT mice (Fig. 1, asterisks). To identify this lipid, we scraped the lipid band from the TLC plate and subjected it to a full-scan LC/MS analysis. We found that several molecular ion peaks had higher intensity in *Nipal4* KO mice than WT mice, especially those with *m/z* 888.9, 916.9, and 944.9 (Fig. 6A). The difference between these *m/z* values is 28, which corresponds to the mass of a hydrocarbon with a carbon chain length of two, suggesting that these molecular ions are derived from the same lipid class.

Next, we subjected the samples to LC/MS/MS analysis in product-ion scanning mode to obtain structural information about these ions. The product ions derived from the precursor ion with *m/z* 916.9 had *m/z* 264.1, 614.4, 642.7, and 898.3 (Fig. 6B). The *m/z* values of 898.3 and 264.1 correspond to the precursor ion subtracting H_2_O and the fragment ion of sphingosine with d18:1 (here, d represents two hydroxyl groups), respectively. The differences between *m/z* 898.3 and 642.7 and between 898.3 and 614.6 are *m/z* 255.6 and 283.7, which correspond to C16:0 and C18:0 FAs, respectively. The *m/z* values 642.7 and 614.6 are the same as those of the product ions of 1-*O*-acylceramides with C26:0 and C24:0 as the *N*-acyl chain, respectively, without the 1-*O*-acyl chain portion (43). These results indicate that the molecular ions with *m/z* 916.9 represent a mixture of 1-*O*-acylceramides with 1-*O*-C18:0/d18:1/C24:0 and 1-*O*-C16:0/d18:1/C26:0. The molecular ions with *m/z* 888.9 and 944.9 are mainly 1-*O*-C16:0/d18:1/C24:0 and 1-*O*-C18:0/d18:1/C26:0, respectively. 1-*O*-Acylceramides were already known to be present in WT mouse epidermis, albeit in small quantities (44). They also exist in human SC and mouse liver, jejunum, and colon (44, 45, 46, 47). However, the production pathway, synthetic enzymes, and physiological functions of 1-*O*-acylceramides in epidermis remain unknown.

Next, we subjected 1-*O*-acylceramides in the epidermis of WT and *Nipal4* KO mice to LC/MS/MS analysis in MRM mode. We detected 186 species with different combinations of 1-*O*-acyl and *N*-acyl chains. The most abundant acyl chains were C24:0 for both the 1-*O*-acyl chains and the *N*-acyl chains in both WT and *Nipal4* KO mice (Fig. 6C, D). All 1-*O*-acylceramide species were increased in *Nipal4* KO mice compared to WT mice (Fig. 6C, D), with an 11-fold increase in total (Fig. 6E).

It has been reported that the levels of 1-*O*-acylceramides are increased in the epidermis of mice with impaired skin barrier formation (such as KO mice for ceramide synthase *Cers3* and *Dgat2*, the latter of which is involved in TG synthesis) (48). To determine whether an increase in 1-*O*-acylceramides is commonly induced by skin barrier defects, we measured 1-*O*-acylceramide levels in mice with impaired skin barrier formation as a result of the KO of genes involved in the synthesis of ω-*O*-acylceramides (FA ω-hydroxylase *Cyp4f39*, FA elongase *Elovl1*, and the acyl-CoA synthase *Fatp4*) via LC/MS/MS. The levels of 1-*O*-acylceramides were increased in *Cyp4f39* KO mice, as in *Nipal4* KO mice, but more modestly (∼4-fold compared to WT mice; Fig. 6E). In contrast, no increase was observed in *Elovl1* KO or *Fatp4* KO mice. These results indicate that the production of 1-*O*-acylceramides is not commonly induced by skin barrier abnormalities but depends on gene KO, and that the increase in 1-*O*-acylceramide levels in *Nipal4* KO mice is particularly prominent.

### Gene expression changes in *Nipal4* KO mice

**Fig. 7:**
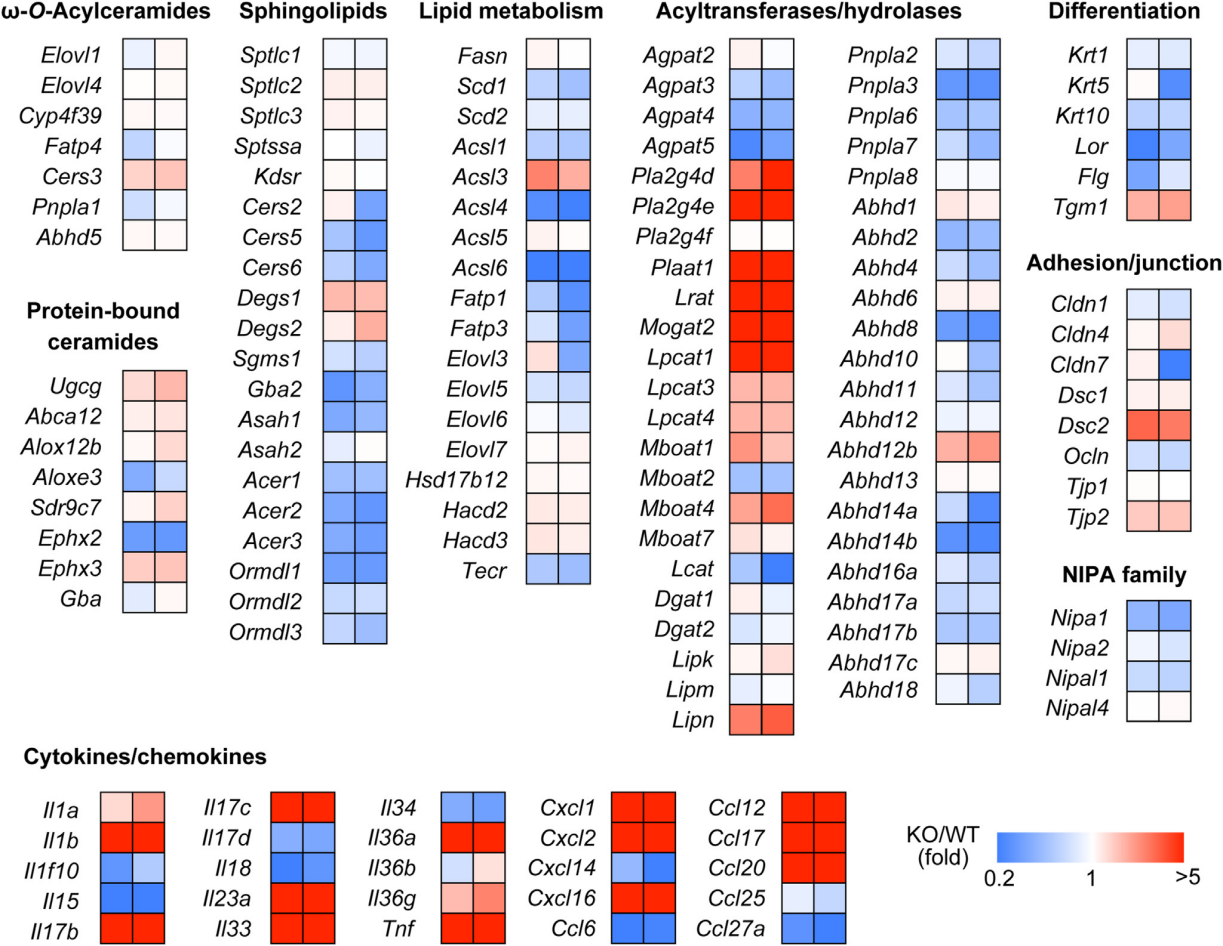
Gene expression changes in the epidermis of *Nipal4* KO mice. Total RNAs were extracted from the epidermis of WT and *Nipal4* KO mice (n = 2 each) on embryonic day 18.5, and comprehensive gene expression analyses were performed by RNA sequencing. The ratio of the number of individual transcripts per million (TPM) for the two *Nipal4* KO mice to the mean number of TPM for the WT mice is shown as a heatmap. A selection of the genes related to ω-*O*-acylceramides, protein-bound ceramides, sphingolipids, other lipids, acyltransferases/hydrolases, differentiation markers, adhesion/junction, the *NIPA* family, and cytokines/chemokines with a TPM of 1 or greater are shown.

To reveal the cause of the observed changes in lipid composition in the epidermis of *Nipal4* KO mice, we compared mRNA levels between WT and *Nipal4* KO mice via RNA sequencing. The expression levels of genes involved in the synthesis or degradation of acylceramides, protein-bound ceramides, and sphingolipids in *Nipal4* KO mice did not differ greatly from those of WT mice (up to a 2.3-fold change; Fig. 7). Of these, we found that dihydroceramide desaturase *Degs1* (producing S-type ceramides from DS-type ceramides) and dihydroceramide 4-hydroxylase *Degs2* (producing P-type ceramides from DS-type ceramides) were weakly upregulated in *Nipal4* KO mice (∼2-fold). It is possible that this causes the increases in S-type and P-type ceramides in *Nipal4* KO mice (Fig. 2A). The expression levels of *NIPA* family genes, genes involved in cell adhesion, and differentiation markers were not greatly altered in *Nipal4* KO mice compared to WT mice (Fig. 7). Among the genes involved in lipid metabolism and those encoding acyltransferases and lipid hydroxylases, the expression levels of the phospholipase family members *Pla2g4d*, *Pla2g4e*, and *Plaat1*, lecithin-retinol acyltransferase *Lrat*, monoacylglycerol acyltransferase *Mogat2*, and lysophospholipid acyltransferase *Lpgat1* were greatly increased in *Nipal4* KO mice. Some cytokines (*Il1a*, *Il1b*, *Il4*, *Il17c*, *Il23a*, *Il33*, *Il36a*, *Il36g*, and *Tnf*) and chemokines (*Cxcl1*, *Cxcl2*, *Cxcl14*, *Cxcl16*, *Ccl12*, *Ccl17*, and *Cxcl20*) were also upregulated in *Nipal4* KO mice.

Among the changes in lipid composition observed in *Nipal4* KO mice (Fig. 2), a decrease in the quantity of ω-*O*-acylceramide EOS may be at least partly responsible for the impaired skin barrier function. ω-*O*-Acylceramides are produced from ω-OH ceramides (O-type ceramides) and linoleic acid-containing TGs by the transacylase PNPLA1. The results of RNA sequencing (Fig. 7) and our previous quantitative real-time RT-PCR (33) showed that *Pnpla1* expression is not reduced in *Nipal4* KO mice. This indicates that the decrease in ω-*O*-acylceramide production in *Nipal4* KO mice is not due to changes in *Pnpla1* expression. Next, to obtain a clue as to the cause of the decrease in ω-*O*-acylceramide production, we examined the Mg^2+^ dependence of the in vitro transacylation reaction by PNPLA1, since NIPAL4 is a magnesium transporter. However, Mg^2+^ and its chelator EDTA did not affect the quantities of the ω-*O*-acylceramide EOS produced (Supplemental Fig. S2), indicating that PNPLA1 does not require Mg^2+^ for catalysis. Thus, the reduced ω-*O*-acylceramide production in *Nipal4* KO mice cannot be explained by changes in *Pnpla1* expression levels or the requirement for Mg^2+^ in the PNPLA1-catalyzed reaction.

## Discussion

The Mg^2+^ ion performs a variety of functions in cells, including acting as a cofactor for various enzymes, providing structural stabilization of phosphate compounds such as ATP, maintaining chromatin structure by binding to DNA, and stabilizing membranes by binding to phospholipids (49, 50). In this study, we performed a comprehensive analysis of the epidermal lipids in *Nipal4* KO mice to clarify the physiological significance of Mg^2+^ in skin barrier formation. We found changes in ceramide composition (including decreases in ω-*O*-acylceramides and increases in ω-OH ceramides), increases in ω-OH Glc-ceramides, TGs, FFAs, and 1-*O*-acylceramides (Fig. 2, Fig. 3, Fig. 4, Fig. 5, Fig. 6), and decreases in OAHFAs containing C18:2 FA (Fig. 5D). Of these changes, at least the decreases in ω-*O*-acylceramides may be related to abnormal skin barrier function in *Nipal4* KO mice. ω-*O*-Acylceramides are required for the formation and maintenance of lipid lamellae (10, 11), and *Nipal4* KO mice indeed show impaired lipid lamella formation (33). However, changes in the levels of other lipids may also be partially responsible for skin barrier abnormalities.

**Fig. 8:**
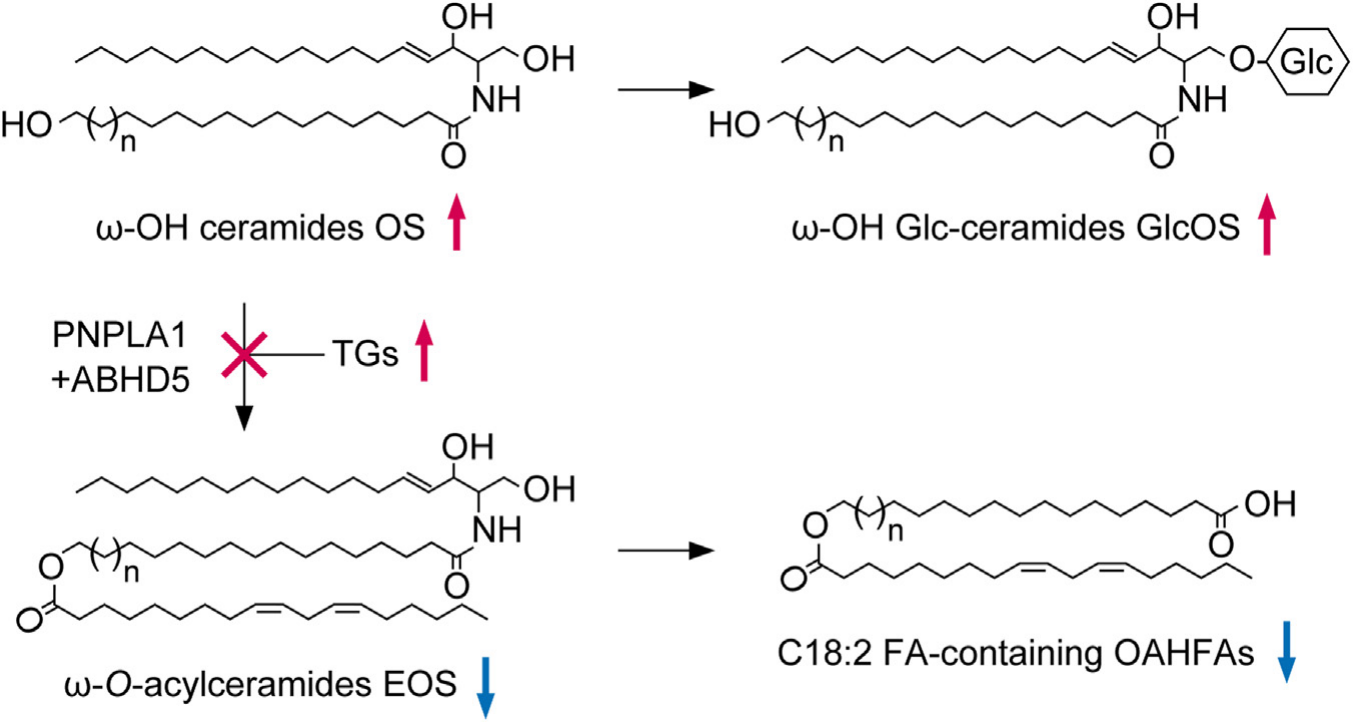
Model of altered lipid composition in *Nipal4* KO mice. In *Nipal4* KO mice, the transacylation reaction by PNPLA1 and ABHD5 is impaired, resulting in decreases in the levels of the ω-*O*-acylceramide EOS and its degradation products, C18:2 FA-containing OAHFAs. In contrast, the substrate of the transacylation reaction, ω-OH ceramide OS and its derivative ω-OH Glc-ceramide GlcOS, accumulate. Red and blue arrows indicate increases and decreases in *Nipal4* KO mice, respectively. OAHFAs, O-acyl-omega-hydroxy fatty acids.

ω-*O*-Acylceramides are produced by the transfer of linoleic acid in TGs to ω-OH ceramides via a transacylation reaction (41). The present results, decreases in the product (ω-*O*-acylceramides) and increases in the precursors (ω-OH ceramides and TGs), suggest that the transacylation reaction is somehow impaired in *Nipal4* KO mice (Fig. 8). This transacylation reaction is catalyzed by the transacylase PNPLA1 and facilitated by the lipid droplet protein ABHD5 (41, 51). TGs, the source of linoleic acid for ω-*O*-acylceramides, are stored in lipid droplets, and ABHD5 is thought to recruit PNPLA1 to the interface between the endoplasmic reticulum (ER) and lipid droplets to facilitate TG utilization by PNPLA1 (51). However, the expression levels of these genes differed little between the WT and *Nipal4* KO mice (Fig. 7) (33). In addition, Mg^2+^ was not required in the transacylation reaction (Supplemental Fig. S2). Therefore, the reason for the impaired transacylation reaction in *Nipal4* KO mice is still unknown, but we speculate that it may involve a change in the ER membrane state (see below). GlcOS and C18:2 FA-containing OAHFAs are produced by glucosylation of OS and degradation of EOS, respectively. Therefore, the increase in GlcOS and decrease in C18:2 FA-containing OAHFAs in *Nipal4* KO mice (Figs. 3 and 5) can be explained by an increase in OS and a decrease in EOS, respectively (Fig. 8).

The epidermis is composed of four layers: stratum basale, stratum spinosum, stratum granulosum, and SC (in order from the inside outwards). Keratinocytes proliferate in the stratum basale and migrate outward while differentiating into cells with respective layer-specific properties and morphology. The lipids constituting lipid lamellae are synthesized mainly in the stratum granulosum and first stored in lamellar bodies, then released into the extracellular space near the boundary between the stratum granulosum and SC. Intracellular Mg^2+^ concentrations increase in keratinocytes in a NIPAL4-dependent manner during differentiation (33). Considering the wide range of Mg^2+^ functions, many cellular events are likely to be affected in *Nipal4* KO mice, among which is the stabilization of the ER membrane through the binding of Mg^2+^ to phospholipids (discussed below). FAs are classified according to chain length into long-chain FAs (C11–C20), very-long-chain FAs (≥C21), and ultra-long-chain (ULC) FAs (≥C26) (52). Of these, ULCFAs are present only in a few tissues, such as epidermis, brain, and retina (8, 52). Epidermal ULCFAs comprise the ω-OH C30–36 FAs constituting ω-OH ceramides, ω-*O*-acylceramides, and protein-bound ceramides (8, 9, 34). The ω-OH ULCFAs are produced in the stratum granulosum via the elongation of long-chain acyl-CoAs to ULC acyl-CoAs by FA elongases (ELOVL1 and ELOVL4), CoA removal, and ω-hydroxylation by FA ω-hydroxylase (human CYP4F22/mouse CYP4F39) (8, 10, 53, 54, 55). In general, lipid synthesis takes place in the cytosolic leaflet of the lipid bilayer of the ER membrane, with the polar groups of the lipids exposed to the cytosol and the acyl groups located within the cytosolic leaflet, and this should also be the case for ω-*O*-acylceramide synthesis. During ω-*O*-acylceramide synthesis, the carboxyl group at the C1 position of the ω-OH ULCFAs undergoes CoA addition by the acyl-CoA synthase FATP4 and amide bond formation with a long-chain base by the ceramide synthase CERS3 on the cytosol surface of the ER membrane. Similarly, the hydroxyl group at the ω-position of the ω-OH ULCFA forms an ester bond with linoleic acid from TGs on the cytosol surface of the ER membrane (51). Reactions at both ends of the ω-OH ULCFAs at the cytosol surface of the ER membrane mean that the C30–36 ULC acyl moieties are bent in the lipid bilayer. Since such bending of the acyl chains is likely to destabilize the ER membrane, we speculate that the increase in Mg^2+^ concentration caused by NIPAL4 stabilizes the ER membrane. Insufficient Mg^2+^ supply due to *Nipal4* deficiency may cause changes in the physical properties of the ER membrane, affecting lipid synthesis (such as reduced FA elongation and ω-*O*-acylceramide synthesis and increased TG and 1-*O*-acylceramide synthesis). The linoleic acid moiety of ω-*O*-acylceramides is important for the conversion of ω-*O*-acylceramides to protein-bound ceramides (56). During the conversion, the linoleic acid moiety undergoes modification to become epoxy-enone, which then binds to corneocyte surface proteins (12, 13). However, we found compositional changes in the *O*-acyl chain of ω-*O*-acylceramides, *that is*, a decrease in linoleic acid and increases in other FAs, in *Nipal4* KO mice (Fig. 2E). Although the exact cause is unknown, it is possible that the change in physical properties of the ER membrane also alters the substrate specificity of PNPLA1 or the conformation of TGs.

Although 1-*O*-acylceramide and ω-*O*-acylceramide are both categorized as acylceramides, they are structurally quite different. In ω-*O*-acylceramides, the three hydrophobic chains (long-chain base, ω-OH FA, and linoleic acid) can extend to become linear. Molecular dynamics simulations indeed predict that ω-*O*-acylceramides span the layers as an extended structure in lipid lamellae (57), which may contribute to stabilization of the lipid lamellae. In contrast, the three hydrophobic chains of 1-*O*-acylceramides (a long-chain base and two FAs) are T-shaped and cannot function to stabilize the lipid lamellae (58). The structure of 1-*O*-acylceramides is similar to that of TGs. Production of 1-*O*-acylceramides is enhanced in the liver of mice fed a high-fat diet, and they are stored in lipid droplets like TGs (45). It has been reported that the acyl-CoA synthase ACSL5, ceramide synthase CERS2, and diacylglycerol acyltransferase DGAT2 are involved in the production of 1-*O*-acylceramides (45). However, analyses of KO mice of these genes have shown that they are not involved in the production of 1-*O*-acylceramides, at least in the epidermis (48). Therefore, the molecular mechanism of 1-*O*-acylceramide production in the epidermis remains unknown. However, since we found that the mRNA levels of some acyltransferases were increased in *Nipal4* KO mice (Fig. 7), it is possible that one of these is involved in 1-*O*-acylceramide production in the epidermis.

In this study, we found that the levels and composition of several lipids were altered in *Nipal4* KO mouse epidermis compared to WT mouse epidermis. This indicates that a differentiation-dependent increase in Mg^2+^ concentration in keratinocytes is necessary for the production of the lipids that constitute normal lipid lamellae. It future, physical and morphological analyses and molecular dynamics simulations are needed for elucidation of the exact role of Mg^2+^ in skin barrier formation.

## Data Availability

All data are included in the article.

## Supplemental data

This article contains supplemental data.

## Conflict of interest

The authors declare that they have no conflicts of interest with the contents of this article.
