# Supplementary material for: Alteration of epidermal lipid composition as a result of deficiency in the magnesium transporter Nipal4
Source: J Lipid Res. 2024 Apr 29;65(6):100550. doi: 10.1016/j.jlr.2024.100550 (PMC11153242; doi:10.1016/j.jlr.2024.100550)
Supplement: Supplemental Data [file mmc1.docx]

**Supplemental DATA**

**Alteration of epidermal lipid composition as a result of deficiency in the magnesium transporter *Nipal4***

Marino Yamaji, Yusuke Ohno, Madoka Shimada, Akio Kihara

**
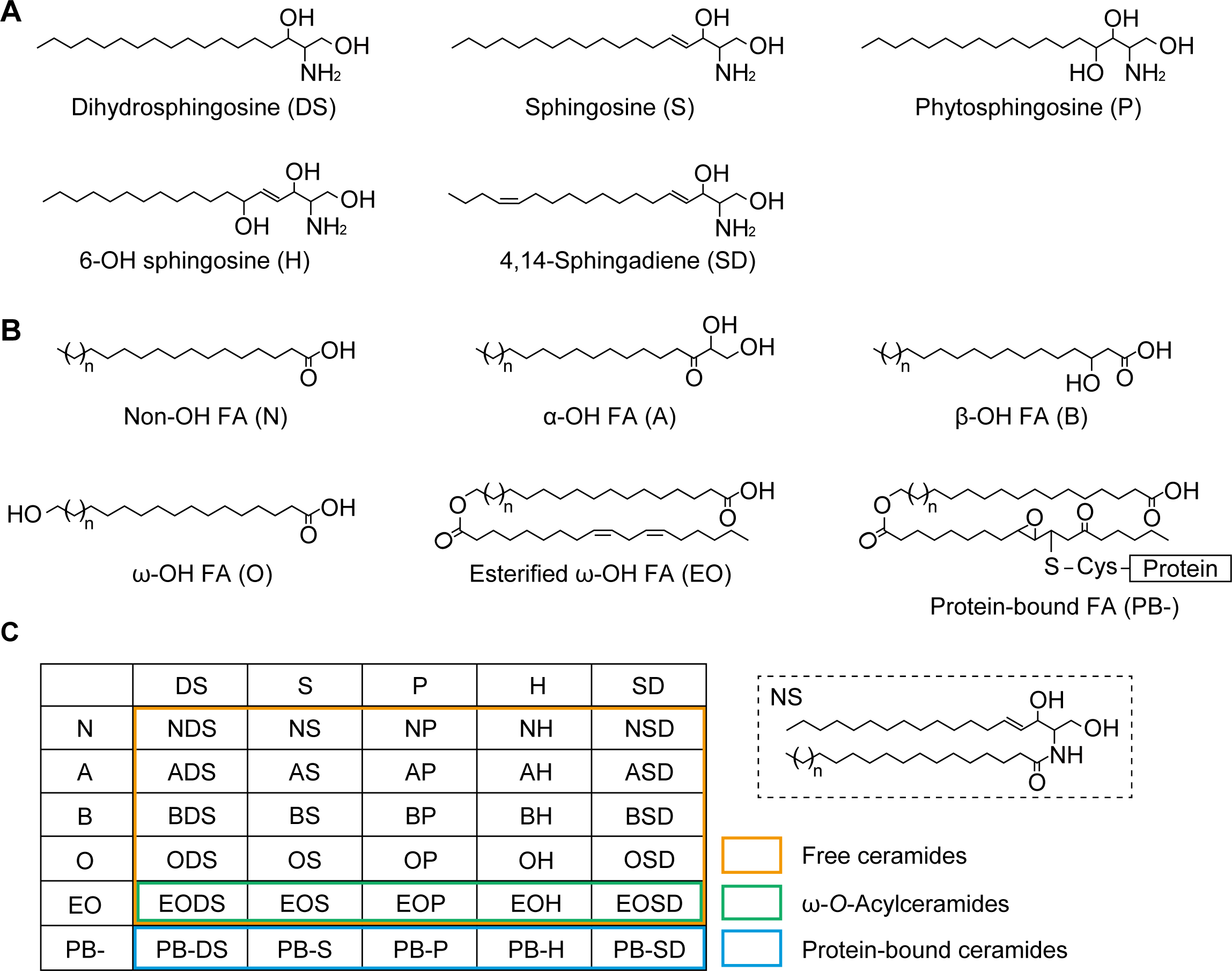
**

**Supplemental Figure S1. Ceramide classes in mammals**

The structures of the long-chain bases (A) and FAs (B) that constitute mammalian ceramides, the nomenclature of each ceramide class (C), and the structure of NS (C).

**
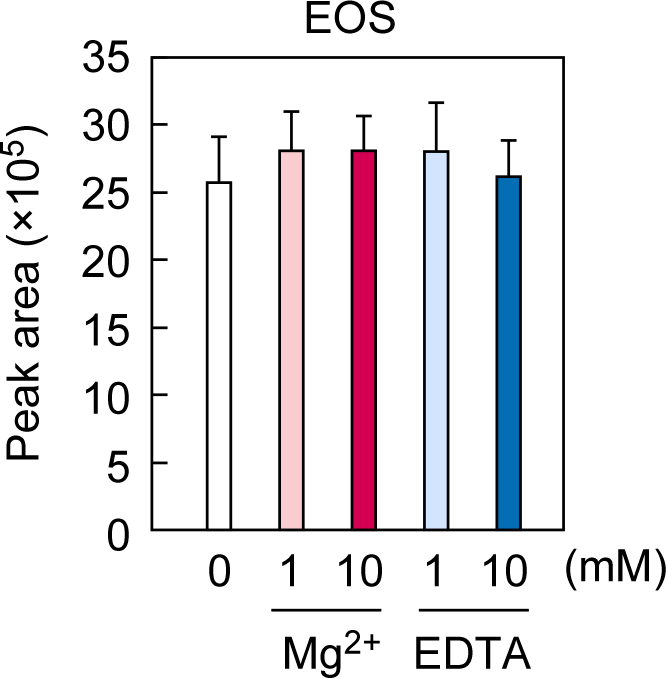
**

**Supplemental Figure S2. Transacylation by PNPLA1 does not require Mg^2+^**

PNPLA1 was translated *in vitro* using wheat germ lysate in the presence of phosphatidylcholine-based liposomes containing TGs and C30:0 ω-OH ceramide. The resulting proteoliposomes were incubated for 1 h at 37°C in the presence of 0, 1, or 10 mM Mg^2+^ or EDTA. Lipids were extracted, and the C30:0 acylceramide EOS was quantified via LC/MS/MS.

**Supplemental Table S1.** MRM settings for detection of Glc-ceramide species in LC/MS/MS analysis.

| Class | *N*-acyl | Precursor ions (Q1) | | Product ion (Q3) | Collision energy (eV) |
| --- | --- | --- | --- | --- | --- |
|  |  | [M + H]^+^ | [M + H – H_2_O]^+^ |  |  |
| GlcNS | C16:0 |  | 682.5 | 264.3 | 40 |
| GlcNS | C17:0 |  | 696.5 | 264.3 | 40 |
| GlcNS | C18:0 |  | 710.6 | 264.3 | 40 |
| GlcNS | C19:0 |  | 724.6 | 264.3 | 40 |
| GlcNS | C20:0 |  | 738.6 | 264.3 | 40 |
| GlcNS | C21:0 |  | 752.6 | 264.3 | 40 |
| GlcNS | C22:0 |  | 766.6 | 264.3 | 40 |
| GlcNS | C23:1 |  | 778.6 | 264.3 | 40 |
| GlcNS | C23:0 |  | 780.6 | 264.3 | 40 |
| GlcNS | C24:1 |  | 792.6 | 264.3 | 40 |
| GlcNS | C24:0 |  | 794.6 | 264.3 | 45 |
| GlcNS | C25:1 |  | 806.6 | 264.3 | 45 |
| GlcNS | C25:0 |  | 808.6 | 264.3 | 45 |
| GlcNS | C26:1 |  | 820.7 | 264.3 | 45 |
| GlcNS | C26:0 |  | 822.7 | 264.3 | 45 |
| GlcNS | C27:1 |  | 834.7 | 264.3 | 45 |
| GlcNS | C27:0 |  | 836.7 | 264.3 | 45 |
| GlcNS | C28:1 |  | 848.7 | 264.3 | 45 |
| GlcNS | C28:0 |  | 850.7 | 264.3 | 45 |
| GlcNS | C29:1 |  | 862.7 | 264.3 | 45 |
| GlcNS | C29:0 |  | 864.7 | 264.3 | 45 |
| GlcNS | C30:1 |  | 876.7 | 264.3 | 50 |
| GlcNS | C30:0 |  | 878.7 | 264.3 | 50 |
| GlcNDS | C16:0 | 702.6 |  | 284.3 | 40 |
| GlcNDS | C17:0 | 716.6 |  | 284.3 | 40 |
| GlcNDS | C18:0 | 730.7 |  | 284.3 | 40 |
| GlcNDS | C19:0 | 744.7 |  | 284.3 | 40 |
| GlcNDS | C20:0 | 758.7 |  | 284.3 | 40 |
| GlcNDS | C21:0 | 772.7 |  | 284.3 | 40 |
| GlcNDS | C22:0 | 786.7 |  | 284.3 | 40 |
| GlcNDS | C23:1 | 798.7 |  | 284.3 | 40 |
| GlcNDS | C23:0 | 800.7 |  | 284.3 | 40 |
| GlcNDS | C24:1 | 812.7 |  | 284.3 | 40 |
| GlcNDS | C24:0 | 814.7 |  | 284.3 | 45 |
| GlcNDS | C25:1 | 826.7 |  | 284.3 | 45 |
| GlcNDS | C25:0 | 828.7 |  | 284.3 | 45 |
| GlcNDS | C26:1 | 840.8 |  | 284.3 | 45 |
| GlcNDS | C26:0 | 842.8 |  | 284.3 | 45 |
| GlcNDS | C27:1 | 854.8 |  | 284.3 | 45 |
| GlcNDS | C27:0 | 856.8 |  | 284.3 | 45 |
| GlcNDS | C28:1 | 868.8 |  | 284.3 | 45 |
| GlcNDS | C28:0 | 870.8 |  | 284.3 | 45 |
| GlcNDS | C29:1 | 882.8 |  | 284.3 | 45 |
| GlcNDS | C29:0 | 884.8 |  | 284.3 | 45 |
| GlcNDS | C30:1 | 896.8 |  | 284.3 | 50 |
| GlcNDS | C30:0 | 898.8 |  | 284.3 | 50 |
| GlcNP | C16:0 | 718.7 |  | 300.3 | 40 |
| GlcNP | C17:0 | 732.7 |  | 300.3 | 40 |
| GlcNP | C18:0 | 746.7 |  | 300.3 | 40 |
| GlcNP | C19:0 | 760.7 |  | 300.3 | 40 |
| GlcNP | C20:0 | 774.7 |  | 300.3 | 40 |
| GlcNP | C21:0 | 788.7 |  | 300.3 | 40 |
| GlcNP | C22:0 | 802.8 |  | 300.3 | 40 |
| GlcNP | C23:1 | 814.8 |  | 300.3 | 40 |
| GlcNP | C23:0 | 816.8 |  | 300.3 | 40 |
| GlcNP | C24:1 | 828.8 |  | 300.3 | 40 |
| GlcNP | C24:0 | 830.8 |  | 300.3 | 45 |
| GlcNP | C25:1 | 842.8 |  | 300.3 | 45 |
| GlcNP | C25:0 | 844.8 |  | 300.3 | 45 |
| GlcNP | C26:1 | 856.8 |  | 300.3 | 45 |
| GlcNP | C26:0 | 858.8 |  | 300.3 | 45 |
| GlcNP | C27:1 | 870.8 |  | 300.3 | 45 |
| GlcNP | C27:0 | 872.8 |  | 300.3 | 45 |
| GlcNP | C28:1 | 884.9 |  | 300.3 | 45 |
| GlcNP | C28:0 | 886.9 |  | 300.3 | 45 |
| GlcNP | C29:1 | 898.9 |  | 300.3 | 45 |
| GlcNP | C29:0 | 900.9 |  | 300.3 | 45 |
| GlcNP | C30:1 | 912.9 |  | 300.3 | 50 |
| GlcNP | C30:0 | 914.9 |  | 300.3 | 50 |
| GlcAS | C16:0 |  | 698.5 | 264.3 | 40 |
| GlcAS | C17:0 |  | 712.5 | 264.3 | 40 |
| GlcAS | C18:0 |  | 726.6 | 264.3 | 40 |
| GlcAS | C19:0 |  | 740.6 | 264.3 | 40 |
| GlcAS | C20:0 |  | 754.6 | 264.3 | 40 |
| GlcAS | C21:0 |  | 768.6 | 264.3 | 40 |
| GlcAS | C22:0 |  | 782.6 | 264.3 | 40 |
| GlcAS | C23:1 |  | 794.6 | 264.3 | 40 |
| GlcAS | C23:0 |  | 796.6 | 264.3 | 40 |
| GlcAS | C24:1 |  | 808.6 | 264.3 | 40 |
| GlcAS | C24:0 |  | 810.6 | 264.3 | 45 |
| GlcAS | C25:1 |  | 822.6 | 264.3 | 45 |
| GlcAS | C25:0 |  | 824.6 | 264.3 | 45 |
| GlcAS | C26:1 |  | 836.7 | 264.3 | 45 |
| GlcAS | C26:0 |  | 838.7 | 264.3 | 45 |
| GlcAS | C27:1 |  | 850.7 | 264.3 | 45 |
| GlcAS | C27:0 |  | 852.7 | 264.3 | 45 |
| GlcAS | C28:1 |  | 864.7 | 264.3 | 45 |
| GlcAS | C28:0 |  | 866.7 | 264.3 | 45 |
| GlcAS | C29:1 |  | 878.7 | 264.3 | 45 |
| GlcAS | C29:0 |  | 880.7 | 264.3 | 45 |
| GlcAS | C30:1 |  | 892.7 | 264.3 | 50 |
| GlcAS | C30:0 |  | 894.7 | 264.3 | 50 |
| GlcADS | C16:0 | 718.6 |  | 284.3 | 40 |
| GlcADS | C17:0 | 732.6 |  | 284.3 | 40 |
| GlcADS | C18:0 | 746.7 |  | 284.3 | 40 |
| GlcADS | C19:0 | 760.7 |  | 284.3 | 40 |
| GlcADS | C20:0 | 774.7 |  | 284.3 | 40 |
| GlcADS | C21:0 | 788.7 |  | 284.3 | 40 |
| GlcADS | C22:0 | 802.7 |  | 284.3 | 40 |
| GlcADS | C23:1 | 814.7 |  | 284.3 | 40 |
| GlcADS | C23:0 | 816.7 |  | 284.3 | 40 |
| GlcADS | C24:1 | 828.7 |  | 284.3 | 40 |
| GlcADS | C24:0 | 830.7 |  | 284.3 | 45 |
| GlcADS | C25:1 | 842.7 |  | 284.3 | 45 |
| GlcADS | C25:0 | 844.7 |  | 284.3 | 45 |
| GlcADS | C26:1 | 856.8 |  | 284.3 | 45 |
| GlcADS | C26:0 | 858.8 |  | 284.3 | 45 |
| GlcADS | C27:1 | 870.8 |  | 284.3 | 45 |
| GlcADS | C27:0 | 872.8 |  | 284.3 | 45 |
| GlcADS | C28:1 | 884.8 |  | 284.3 | 45 |
| GlcADS | C28:0 | 886.8 |  | 284.3 | 45 |
| GlcADS | C29:1 | 898.8 |  | 284.3 | 45 |
| GlcADS | C29:0 | 900.8 |  | 284.3 | 45 |
| GlcADS | C30:1 | 912.8 |  | 284.3 | 50 |
| GlcADS | C30:0 | 914.8 |  | 284.3 | 50 |
| GlcAP | C16:0 | 734.7 |  | 300.3 | 40 |
| GlcAP | C17:0 | 748.7 |  | 300.3 | 40 |
| GlcAP | C18:0 | 762.7 |  | 300.3 | 40 |
| GlcAP | C19:0 | 776.7 |  | 300.3 | 40 |
| GlcAP | C20:0 | 790.7 |  | 300.3 | 40 |
| GlcAP | C21:0 | 804.7 |  | 300.3 | 40 |
| GlcAP | C22:0 | 818.8 |  | 300.3 | 40 |
| GlcAP | C23:1 | 830.8 |  | 300.3 | 40 |
| GlcAP | C23:0 | 832.8 |  | 300.3 | 40 |
| GlcAP | C24:1 | 844.8 |  | 300.3 | 40 |
| GlcAP | C24:0 | 846.8 |  | 300.3 | 45 |
| GlcAP | C25:1 | 858.8 |  | 300.3 | 45 |
| GlcAP | C25:0 | 860.8 |  | 300.3 | 45 |
| GlcAP | C26:1 | 872.8 |  | 300.3 | 45 |
| GlcAP | C26:0 | 874.8 |  | 300.3 | 45 |
| GlcAP | C27:1 | 886.8 |  | 300.3 | 45 |
| GlcAP | C27:0 | 888.8 |  | 300.3 | 45 |
| GlcAP | C28:1 | 900.9 |  | 300.3 | 45 |
| GlcAP | C28:0 | 902.9 |  | 300.3 | 45 |
| GlcAP | C29:1 | 914.9 |  | 300.3 | 45 |
| GlcAP | C29:0 | 916.9 |  | 300.3 | 45 |
| GlcAP | C30:1 | 928.9 |  | 300.3 | 50 |
| GlcAP | C30:0 | 930.9 |  | 300.3 | 50 |
| GlcOS | C28:1 | 864.7 | 882.7 | 264.3 | 45 |
| GlcOS | C28:0 | 866.7 | 884.7 | 264.3 | 45 |
| GlcOS | C29:1 | 878.7 | 896.7 | 264.3 | 45 |
| GlcOS | C29:0 | 880.7 | 898.7 | 264.3 | 45 |
| GlcOS | C30:1 | 892.7 | 910.7 | 264.3 | 50 |
| GlcOS | C30:0 | 894.7 | 912.7 | 264.3 | 50 |
| GlcOS | C31:1 | 906.7 | 924.7 | 264.3 | 50 |
| GlcOS | C31:0 | 908.7 | 926.7 | 264.3 | 50 |
| GlcOS | C32:1 | 920.8 | 938.8 | 264.3 | 55 |
| GlcOS | C32:0 | 922.8 | 940.8 | 264.3 | 55 |
| GlcOS | C33:1 | 934.8 | 952.8 | 264.3 | 55 |
| GlcOS | C33:0 | 936.8 | 954.8 | 264.3 | 55 |
| GlcOS | C34:1 | 948.8 | 966.8 | 264.3 | 55 |
| GlcOS | C34:0 | 950.8 | 968.8 | 264.3 | 55 |
| GlcOS | C35:1 | 962.8 | 980.8 | 264.3 | 55 |
| GlcOS | C35:0 | 964.8 | 982.8 | 264.3 | 55 |
| GlcOS | C36:1 | 976.8 | 994.8 | 264.3 | 55 |
| GlcOS | C36:0 | 978.8 | 996.8 | 264.3 | 55 |
| GlcEOS | C28:1 | 1127.0 | 1145.0 | 264.3 | 45 |
| GlcEOS | C28:0 | 1129.0 | 1147.0 | 264.3 | 45 |
| GlcEOS | C29:1 | 1141.0 | 1159.0 | 264.3 | 50 |
| GlcEOS | C29:0 | 1143.0 | 1161.0 | 264.3 | 50 |
| GlcEOS | C30:1 | 1155.0 | 1173.0 | 264.3 | 50 |
| GlcEOS | C30:0 | 1157.0 | 1175.0 | 264.3 | 50 |
| GlcEOS | C31:1 | 1169.0 | 1187.0 | 264.3 | 50 |
| GlcEOS | C31:0 | 1171.0 | 1189.0 | 264.3 | 50 |
| GlcEOS | C32:1 | 1183.0 | 1201.0 | 264.3 | 50 |
| GlcEOS | C32:0 | 1185.0 | 1203.0 | 264.3 | 50 |
| GlcEOS | C33:1 | 1197.0 | 1215.0 | 264.3 | 50 |
| GlcEOS | C33:0 | 1199.0 | 1217.0 | 264.3 | 50 |
| GlcEOS | C34:1 | 1211.1 | 1229.1 | 264.3 | 50 |
| GlcEOS | C34:0 | 1213.1 | 1231.1 | 264.3 | 50 |
| GlcEOS | C35:1 | 1225.1 | 1243.1 | 264.3 | 50 |
| GlcEOS | C35:0 | 1227.1 | 1245.1 | 264.3 | 50 |
| GlcEOS | C36:1 | 1239.1 | 1257.1 | 264.3 | 55 |
| GlcEOS | C36:0 | 1241.1 | 1259.1 | 264.3 | 55 |
| GlcEODS | C28:1 | 1129.1 | 1147.1 | 284.3 | 45 |
| GlcEODS | C28:0 | 1131.1 | 1149.1 | 284.3 | 45 |
| GlcEODS | C29:1 | 1143.1 | 1161.1 | 284.3 | 50 |
| GlcEODS | C29:0 | 1145.1 | 1163.1 | 284.3 | 50 |
| GlcEODS | C30:1 | 1157.1 | 1175.1 | 284.3 | 50 |
| GlcEODS | C30:0 | 1159.1 | 1177.1 | 284.3 | 50 |
| GlcEODS | C31:1 | 1171.1 | 1189.1 | 284.3 | 50 |
| GlcEODS | C31:0 | 1173.1 | 1191.1 | 284.3 | 50 |
| GlcEODS | C32:1 | 1185.1 | 1203.1 | 284.3 | 50 |
| GlcEODS | C32:0 | 1187.1 | 1205.1 | 284.3 | 50 |
| GlcEODS | C33:1 | 1199.1 | 1217.1 | 284.3 | 50 |
| GlcEODS | C33:0 | 1201.1 | 1219.1 | 284.3 | 50 |
| GlcEODS | C34:1 | 1213.2 | 1231.2 | 284.3 | 50 |
| GlcEODS | C34:0 | 1215.2 | 1233.2 | 284.3 | 50 |
| GlcEODS | C35:1 | 1227.2 | 1245.2 | 284.3 | 50 |
| GlcEODS | C35:0 | 1229.2 | 1247.2 | 284.3 | 50 |
| GlcEODS | C36:1 | 1241.2 | 1259.2 | 284.3 | 55 |
| GlcEODS | C36:0 | 1243.2 | 1261.2 | 284.3 | 55 |
| GlcEOP | C28:1 | 1145.1 | 1163.1 | 300.3 | 45 |
| GlcEOP | C28:0 | 1147.1 | 1165.1 | 300.3 | 45 |
| GlcEOP | C29:1 | 1159.1 | 1177.1 | 300.3 | 50 |
| GlcEOP | C29:0 | 1161.1 | 1179.1 | 300.3 | 50 |
| GlcEOP | C30:1 | 1173.1 | 1191.1 | 300.3 | 50 |
| GlcEOP | C30:0 | 1175.1 | 1193.1 | 300.3 | 50 |
| GlcEOP | C31:1 | 1187.1 | 1205.1 | 300.3 | 50 |
| GlcEOP | C31:0 | 1189.1 | 1207.1 | 300.3 | 50 |
| GlcEOP | C32:1 | 1201.1 | 1219.1 | 300.3 | 50 |
| GlcEOP | C32:0 | 1203.1 | 1221.1 | 300.3 | 50 |
| GlcEOP | C33:1 | 1215.1 | 1233.1 | 300.3 | 50 |
| GlcEOP | C33:0 | 1217.1 | 1235.1 | 300.3 | 50 |
| GlcEOP | C34:1 | 1229.2 | 1247.2 | 300.3 | 50 |
| GlcEOP | C34:0 | 1231.2 | 1249.2 | 300.3 | 50 |
| GlcEOP | C35:1 | 1243.2 | 1261.2 | 300.3 | 50 |
| GlcEOP | C35:0 | 1245.2 | 1263.2 | 300.3 | 50 |
| GlcEOP | C36:1 | 1257.2 | 1275.2 | 300.3 | 55 |
| GlcEOP | C36:0 | 1259.2 | 1277.2 | 300.3 | 55 |

**Supplemental Table S2.** MRM settings for detection of TG species in LC/MS/MS analysis.

| TG species | Precursor ion (Q1) | Product ion (Q3) | Collision energy (eV) |
| --- | --- | --- | --- |
|  | [M+NH_4_]^+^ |  |  |
| C16:0-C32:1 | 822.7 | 549.5 | 20 |
| C16:0-C32:0 | 824.7 | 551.5 | 20 |
| C16:0-C34:3 | 846.8 | 573.5 | 20 |
| C16:0-C34:2 | 848.8 | 575.5 | 20 |
| C16:0-C34:1 | 850.8 | 577.5 | 20 |
| C16:0-C34:0 | 852.8 | 579.5 | 20 |
| C16:0-C36:3 | 874.8 | 601.6 | 20 |
| C16:0-C36:2 | 876.8 | 603.5 | 20 |
| C16:0-C36:1 | 878.8 | 605.5 | 20 |
| C16:0-C38:1 | 906.7 | 633.5 | 20 |
| C18:1-C32:1 | 848.8 | 549.5 | 20 |
| C18:1-C32:0 | 850.8 | 551.5 | 20 |
| C18:1-C34:2 | 874.8 | 575.6 | 20 |
| C18:1-C34:1 | 876.8 | 577.5 | 20 |
| C18:1-C34:0 | 878.7 | 579.5 | 20 |
| C18:1-C36:3 | 900.7 | 601.5 | 20 |
| C18:1-C36:2 | 902.7 | 603.5 | 20 |
| C18:1-C36:1 | 904.7 | 605.5 | 20 |
| C18:1-C36:0 | 906.7 | 607.5 | 20 |
| C18:1-C38:1 | 932.8 | 633.5 | 20 |
| C18:2-C32:1 | 846.8 | 549.5 | 20 |
| C18:2-C32:0 | 848.8 | 551.5 | 20 |
| C18:2-C34:2 | 872.8 | 575.5 | 20 |
| C18:2-C34:1 | 874.8 | 577.6 | 20 |
| C18:2-C34:0 | 876.7 | 579.5 | 20 |
| C18:2-C36:3 | 898.8 | 601.6 | 20 |
| C18:2-C36:2 | 900.7 | 603.5 | 20 |
| C18:2-C36:1 | 902.7 | 605.5 | 20 |
| C18:2-C36:0 | 904.7 | 607.5 | 20 |
| C18:2-C38:1 | 930.8 | 633.5 | 20 |
| C18:1-C15:0-C15:0-*d*_5_* | 827.7 | 528.5 | 20 |

*Internal standard

**Supplemental Table S3.** MRM settings for detection of FFA species in LC/MS/MS analysis.

| FA | Precursor ion (Q1) | Product ion (Q3) | Collision energy (eV) |
| --- | --- | --- | --- |
|  | [M + AMPP]^+^ |  |  |
| C20:0 | 479.6 | 239.0 | 50 |
| C22:0 | 507.6 | 239.0 | 50 |
| C24:0 | 535.7 | 239.0 | 50 |
| C26:0 | 563.7 | 239.0 | 50 |
| C28:0 | 591.7 | 239.0 | 50 |
| C30:0 | 619.7 | 239.0 | 50 |
| C32:0 | 647.8 | 239.0 | 50 |
| C34:0 | 675.8 | 239.0 | 50 |
| C36:0 | 703.8 | 239.0 | 50 |
| *d*_31_-C16:0* | 454.4 | 243.0 | 50 |

*Internal standard

**Supplemental Table S4.** MRM settings for detection of OAHFA species in LC/MS/MS analysis.

| ω*-O*-acyl | FA | Precursor ion (Q1) | Product ion (Q3) | Collision energy (eV) |
| --- | --- | --- | --- | --- |
|  |  | [M + AMPP]^+^ |  |  |
| C16:1 | ω-OH C30:1 | 869.7 | 615.2 | 15 |
| C16:1 | ω-OH C30:0 | 871.7 | 617.2 | 15 |
| C16:1 | ω-OH C31:1 | 883.7 | 629.2 | 15 |
| C16:1 | ω-OH C31:0 | 885.7 | 631.2 | 15 |
| C16:1 | ω-OH C32:1 | 897.7 | 643.2 | 15 |
| C16:1 | ω-OH C32:0 | 899.7 | 645.3 | 15 |
| C16:1 | ω-OH C33:1 | 911.7 | 657.3 | 15 |
| C16:1 | ω-OH C33:0 | 913.7 | 659.3 | 15 |
| C16:1 | ω-OH C34:1 | 925.7 | 671.3 | 15 |
| C16:1 | ω-OH C34:0 | 927.8 | 673.3 | 15 |
| C16:1 | ω-OH C35:1 | 939.8 | 685.3 | 15 |
| C16:1 | ω-OH C35:0 | 941.8 | 687.3 | 15 |
| C16:1 | ω-OH C36:1 | 953.8 | 699.3 | 15 |
| C16:1 | ω-OH C36:0 | 955.8 | 701.3 | 15 |
| C18:1 | ω-OH C30:1 | 897.7 | 615.2 | 15 |
| C18:1 | ω-OH C30:0 | 899.7 | 617.2 | 15 |
| C18:1 | ω-OH C31:1 | 911.7 | 629.2 | 15 |
| C18:1 | ω-OH C31:0 | 913.7 | 631.2 | 15 |
| C18:1 | ω-OH C32:1 | 925.7 | 643.2 | 15 |
| C18:1 | ω-OH C32:0 | 927.7 | 645.3 | 15 |
| C18:1 | ω-OH C33:1 | 939.7 | 657.3 | 15 |
| C18:1 | ω-OH C33:0 | 941.7 | 659.3 | 15 |
| C18:1 | ω-OH C34:1 | 953.7 | 671.3 | 15 |
| C18:1 | ω-OH C34:0 | 955.8 | 673.3 | 15 |
| C18:1 | ω-OH C35:1 | 967.8 | 685.3 | 15 |
| C18:1 | ω-OH C35:0 | 969.8 | 687.3 | 15 |
| C18:1 | ω-OH C36:1 | 981.8 | 699.3 | 15 |
| C18:1 | ω-OH C36:0 | 983.8 | 701.3 | 15 |
| C18:2 | ω-OH C30:1 | 895.7 | 615.2 | 15 |
| C18:2 | ω-OH C30:0 | 897.7 | 617.2 | 15 |
| C18:2 | ω-OH C31:1 | 909.7 | 629.2 | 15 |
| C18:2 | ω-OH C31:0 | 911.7 | 631.2 | 15 |
| C18:2 | ω-OH C32:1 | 923.7 | 643.2 | 15 |
| C18:2 | ω-OH C32:0 | 925.7 | 645.3 | 15 |
| C18:2 | ω-OH C33:1 | 937.7 | 657.3 | 15 |
| C18:2 | ω-OH C33:0 | 939.7 | 659.3 | 15 |
| C18:2 | ω-OH C34:1 | 951.7 | 671.3 | 15 |
| C18:2 | ω-OH C34:0 | 953.8 | 673.3 | 15 |
| C18:2 | ω-OH C35:1 | 965.8 | 685.3 | 15 |
| C18:2 | ω-OH C35:0 | 967.8 | 687.3 | 15 |
| C18:2 | ω-OH C36:1 | 979.8 | 699.3 | 15 |
| C18:2 | ω-OH C36:0 | 981.8 | 701.3 | 15 |

**Supplemental Table S5.** MRM settings for detection of 1-*O*-acylceramide species in LC/MS/MS analysis.

| 1-*O-*acyl | *N-*acyl | Precursor ions (Q1) | | Product ion (Q3) | Collision energy (eV) |
| --- | --- | --- | --- | --- | --- |
|  |  | [M + H]^+^ | [M + H – H_2_O]^+^ |  |  |
| C14:0 | C14:0 | 720.7 | 702.7 | 474.5 | 40 |
| C14:0 | C16:0 | 748.7 | 730.7 | 502.5 | 40 |
| C14:0 | C18:0 | 776.7 | 758.7 | 530.5 | 40 |
| C14:0 | C20:0 | 804.8 | 786.8 | 558.6 | 40 |
| C14:0 | C21:0 | 818.8 | 800.8 | 572.6 | 40 |
| C14:0 | C22:0 | 832.8 | 814.8 | 586.6 | 40 |
| C14:0 | C23:0 | 846.8 | 828.8 | 600.6 | 45 |
| C14:0 | C24:1 | 858.9 | 840.9 | 612.6 | 45 |
| C14:0 | C24:0 | 860.8 | 842.8 | 614.6 | 40 |
| C14:0 | C25:0 | 874.8 | 856.8 | 628.6 | 45 |
| C14:0 | C26:1 | 886.9 | 868.9 | 640.7 | 45 |
| C14:0 | C26:0 | 888.9 | 870.9 | 642.7 | 40 |
| C14:0 | C27:0 | 902.8 | 884.8 | 656.6 | 45 |
| C14:0 | C28:0 | 916.9 | 898.9 | 670.7 | 40 |
| C16:0 | C14:0 | 748.7 | 730.7 | 474.5 | 40 |
| C16:0 | C16:0 | 776.7 | 758.7 | 502.5 | 40 |
| C16:0 | C18:0 | 804.8 | 786.8 | 530.5 | 40 |
| C16:0 | C20:0 | 832.8 | 814.8 | 558.6 | 40 |
| C16:0 | C21:0 | 846.8 | 828.8 | 572.6 | 40 |
| C16:0 | C22:0 | 860.8 | 842.8 | 586.6 | 40 |
| C16:0 | C23:0 | 874.8 | 856.8 | 600.6 | 45 |
| C16:0 | C24:1 | 886.9 | 868.9 | 612.6 | 45 |
| C16:0 | C24:0 | 888.9 | 870.9 | 614.6 | 40 |
| C16:0 | C25:0 | 902.8 | 884.8 | 628.6 | 45 |
| C16:0 | C26:1 | 914.9 | 896.9 | 640.7 | 45 |
| C16:0 | C26:0 | 916.9 | 898.9 | 642.7 | 45 |
| C16:0 | C27:0 | 930.8 | 912.8 | 656.6 | 45 |
| C16:0 | C28:0 | 944.9 | 926.9 | 670.7 | 45 |
| C18:0 | C14:0 | 776.8 | 758.8 | 474.5 | 45 |
| C18:0 | C16:0 | 804.8 | 786.8 | 502.5 | 45 |
| C18:0 | C18:0 | 832.8 | 814.8 | 530.5 | 40 |
| C18:0 | C20:0 | 860.8 | 842.8 | 558.6 | 45 |
| C18:0 | C21:0 | 874.8 | 856.8 | 572.6 | 40 |
| C18:0 | C22:0 | 888.9 | 870.9 | 586.6 | 45 |
| C18:0 | C23:0 | 902.9 | 884.9 | 600.6 | 45 |
| C18:0 | C24:1 | 914.9 | 896.9 | 612.6 | 45 |
| C18:0 | C24:0 | 916.9 | 898.9 | 614.6 | 45 |
| C18:0 | C25:0 | 930.9 | 912.9 | 628.6 | 45 |
| C18:0 | C26:1 | 942.9 | 924.9 | 640.7 | 45 |
| C18:0 | C26:0 | 944.9 | 926.9 | 642.7 | 45 |
| C18:0 | C27:0 | 958.9 | 940.9 | 656.6 | 45 |
| C18:0 | C28:0 | 972.9 | 954.9 | 670.7 | 45 |
| C20:0 | C14:0 | 804.8 | 786.8 | 474.5 | 40 |
| C20:0 | C16:0 | 832.8 | 814.8 | 502.5 | 40 |
| C20:0 | C18:0 | 860.8 | 842.8 | 530.5 | 40 |
| C20:0 | C20:0 | 888.9 | 870.9 | 558.6 | 40 |
| C20:0 | C21:0 | 902.8 | 884.8 | 572.6 | 40 |
| C20:0 | C22:0 | 916.9 | 898.9 | 586.6 | 40 |
| C20:0 | C23:0 | 930.9 | 912.9 | 600.6 | 45 |
| C20:0 | C24:1 | 942.9 | 924.9 | 612.6 | 45 |
| C20:0 | C24:0 | 944.9 | 926.9 | 614.6 | 45 |
| C20:0 | C25:0 | 958.9 | 940.9 | 628.6 | 45 |
| C20:0 | C26:1 | 971.0 | 953.0 | 640.7 | 45 |
| C20:0 | C26:0 | 973.0 | 955.0 | 642.7 | 45 |
| C20:0 | C27:0 | 986.9 | 968.9 | 656.6 | 45 |
| C20:0 | C28:0 | 1001.0 | 983.0 | 670.7 | 45 |
| C21:0 | C14:0 | 818.8 | 800.8 | 474.5 | 40 |
| C21:0 | C16:0 | 846.8 | 828.8 | 502.5 | 40 |
| C21:0 | C18:0 | 874.8 | 856.8 | 530.5 | 40 |
| C21:0 | C20:0 | 902.9 | 884.9 | 558.6 | 40 |
| C21:0 | C21:0 | 916.9 | 898.9 | 572.6 | 40 |
| C21:0 | C22:0 | 930.9 | 912.9 | 586.6 | 40 |
| C21:0 | C23:0 | 944.9 | 926.9 | 600.6 | 45 |
| C21:0 | C24:1 | 956.9 | 938.9 | 612.6 | 45 |
| C21:0 | C24:0 | 958.9 | 940.9 | 614.6 | 45 |
| C21:0 | C25:0 | 972.9 | 954.9 | 628.6 | 45 |
| C21:0 | C26:1 | 985.0 | 967.0 | 640.7 | 45 |
| C21:0 | C26:0 | 987.0 | 969.0 | 642.7 | 45 |
| C21:0 | C27:0 | 1001.0 | 983.0 | 656.6 | 45 |
| C21:0 | C28:0 | 1015.0 | 997.0 | 670.7 | 45 |
| C22:0 | C14:0 | 832.8 | 814.8 | 474.5 | 40 |
| C22:0 | C16:0 | 860.8 | 842.8 | 502.5 | 40 |
| C22:0 | C18:0 | 888.9 | 870.9 | 530.5 | 40 |
| C22:0 | C20:0 | 916.9 | 898.9 | 558.6 | 40 |
| C22:0 | C21:0 | 930.9 | 912.9 | 572.6 | 40 |
| C22:0 | C22:0 | 944.9 | 926.9 | 586.6 | 45 |
| C22:0 | C23:0 | 958.9 | 940.9 | 600.6 | 45 |
| C22:0 | C24:1 | 971.0 | 953.0 | 612.6 | 45 |
| C22:0 | C24:0 | 973.0 | 955.0 | 614.6 | 45 |
| C22:0 | C25:0 | 986.9 | 968.9 | 628.6 | 45 |
| C22:0 | C26:1 | 999.0 | 981.0 | 640.7 | 45 |
| C22:0 | C26:0 | 1001.0 | 983.0 | 642.7 | 45 |
| C22:0 | C27:0 | 1014.9 | 996.9 | 656.6 | 45 |
| C22:0 | C28:0 | 1029.0 | 1011.0 | 670.7 | 45 |
| C23:0 | C14:0 | 846.8 | 828.8 | 474.5 | 45 |
| C23:0 | C16:0 | 874.8 | 856.8 | 502.5 | 45 |
| C23:0 | C18:0 | 902.9 | 884.9 | 530.5 | 45 |
| C23:0 | C20:0 | 930.9 | 912.9 | 558.6 | 45 |
| C23:0 | C21:0 | 944.9 | 926.9 | 572.6 | 40 |
| C23:0 | C22:0 | 958.9 | 940.9 | 586.6 | 45 |
| C23:0 | C23:0 | 973.0 | 955.0 | 600.6 | 50 |
| C23:0 | C24:1 | 985.0 | 967.0 | 612.6 | 50 |
| C23:0 | C24:0 | 987.0 | 969.0 | 614.6 | 45 |
| C23:0 | C25:0 | 1001.0 | 983.0 | 628.6 | 50 |
| C23:0 | C26:1 | 1013.0 | 995.0 | 640.7 | 50 |
| C23:0 | C26:0 | 1015.0 | 997.0 | 642.7 | 50 |
| C23:0 | C27:0 | 1029.0 | 1011.0 | 656.6 | 50 |
| C23:0 | C28:0 | 1043.0 | 1025.0 | 670.7 | 50 |
| C24:0 | C14:0 | 860.9 | 842.9 | 474.5 | 40 |
| C24:0 | C16:0 | 888.9 | 870.9 | 502.5 | 40 |
| C24:0 | C18:0 | 916.9 | 898.9 | 530.5 | 40 |
| C24:0 | C20:0 | 944.9 | 926.9 | 558.6 | 45 |
| C24:0 | C21:0 | 958.9 | 940.9 | 572.6 | 40 |
| C24:0 | C22:0 | 973.0 | 955.0 | 586.6 | 45 |
| C24:0 | C23:0 | 987.0 | 969.0 | 600.6 | 45 |
| C24:0 | C24:1 | 999.0 | 981.0 | 612.6 | 45 |
| C24:0 | C24:0 | 1001.0 | 983.0 | 614.6 | 45 |
| C24:0 | C25:0 | 1015.0 | 997.0 | 628.6 | 45 |
| C24:0 | C26:1 | 1027.0 | 1009.0 | 640.7 | 45 |
| C24:0 | C26:0 | 1029.0 | 1011.0 | 642.7 | 45 |
| C24:0 | C27:0 | 1043.0 | 1025.0 | 656.6 | 45 |
| C24:0 | C28:0 | 1057.1 | 1039.1 | 670.7 | 50 |
| C24:1 | C14:0 | 858.9 | 840.9 | 474.5 | 45 |
| C24:1 | C16:0 | 886.9 | 868.9 | 502.5 | 45 |
| C24:1 | C18:0 | 914.9 | 896.9 | 530.5 | 45 |
| C24:1 | C20:0 | 942.9 | 924.9 | 558.6 | 45 |
| C24:1 | C21:0 | 956.9 | 938.9 | 572.6 | 40 |
| C24:1 | C22:0 | 971.0 | 953.0 | 586.6 | 45 |
| C24:1 | C23:0 | 985.0 | 967.0 | 600.6 | 50 |
| C24:1 | C24:1 | 997.0 | 979.0 | 612.6 | 50 |
| C24:1 | C24:0 | 999.0 | 981.0 | 614.6 | 45 |
| C24:1 | C25:0 | 1013.0 | 995.0 | 628.6 | 50 |
| C24:1 | C26:1 | 1025.0 | 1007.0 | 640.7 | 50 |
| C24:1 | C26:0 | 1027.0 | 1009.0 | 642.7 | 50 |
| C24:1 | C27:0 | 1041.0 | 1023.0 | 656.6 | 50 |
| C24:1 | C28:0 | 1055.1 | 1037.1 | 670.7 | 50 |
| C25:0 | C14:0 | 874.9 | 856.9 | 474.5 | 45 |
| C25:0 | C16:0 | 902.9 | 884.9 | 502.5 | 45 |
| C25:0 | C18:0 | 930.9 | 912.9 | 530.5 | 45 |
| C25:0 | C20:0 | 958.9 | 940.9 | 558.6 | 45 |
| C25:0 | C21:0 | 972.9 | 954.9 | 572.6 | 40 |
| C25:0 | C22:0 | 987.0 | 969.0 | 586.6 | 45 |
| C25:0 | C23:0 | 1001.0 | 983.0 | 600.6 | 50 |
| C25:0 | C24:1 | 1013.0 | 995.0 | 612.6 | 45 |
| C25:0 | C24:0 | 1015.0 | 997.0 | 614.6 | 50 |
| C25:0 | C25:0 | 1029.0 | 1011.0 | 628.6 | 50 |
| C25:0 | C26:1 | 1041.0 | 1023.0 | 640.7 | 50 |
| C25:0 | C26:0 | 1043.0 | 1025.0 | 642.7 | 50 |
| C25:0 | C27:0 | 1057.0 | 1039.0 | 656.6 | 50 |
| C25:0 | C28:0 | 1071.1 | 1053.1 | 670.7 | 50 |
| C26:0 | C14:0 | 888.9 | 870.9 | 474.5 | 45 |
| C26:0 | C16:0 | 916.9 | 898.9 | 502.5 | 45 |
| C26:0 | C18:0 | 944.9 | 926.9 | 530.5 | 45 |
| C26:0 | C20:0 | 973.0 | 955.0 | 558.6 | 45 |
| C26:0 | C21:0 | 987.0 | 969.0 | 572.6 | 40 |
| C26:0 | C22:0 | 1001.0 | 983.0 | 586.6 | 45 |
| C26:0 | C23:0 | 1015.0 | 997.0 | 600.6 | 50 |
| C26:0 | C24:1 | 1027.0 | 1009.0 | 612.6 | 50 |
| C26:0 | C24:0 | 1029.0 | 1011.0 | 614.6 | 45 |
| C26:0 | C25:0 | 1043.0 | 1025.0 | 628.6 | 50 |
| C26:0 | C26:1 | 1055.1 | 1037.1 | 640.7 | 50 |
| C26:0 | C26:0 | 1057.1 | 1039.1 | 642.7 | 50 |
| C26:0 | C27:0 | 1071.0 | 1053.0 | 656.6 | 50 |
| C26:0 | C28:0 | 1085.1 | 1067.1 | 670.7 | 50 |
| C26:1 | C14:0 | 886.9 | 868.9 | 474.5 | 45 |
| C26:1 | C16:0 | 914.9 | 896.9 | 502.5 | 45 |
| C26:1 | C18:0 | 942.9 | 924.9 | 530.5 | 45 |
| C26:1 | C20:0 | 971.0 | 953.0 | 558.6 | 45 |
| C26:1 | C21:0 | 985.0 | 967.0 | 572.6 | 40 |
| C26:1 | C22:0 | 999.0 | 981.0 | 586.6 | 45 |
| C26:1 | C23:0 | 1013.0 | 995.0 | 600.6 | 50 |
| C26:1 | C24:1 | 1025.0 | 1007.0 | 612.6 | 50 |
| C26:1 | C24:0 | 1027.0 | 1009.0 | 614.6 | 45 |
| C26:1 | C25:0 | 1041.0 | 1023.0 | 628.6 | 50 |
| C26:1 | C26:1 | 1053.0 | 1035.0 | 640.7 | 50 |
| C26:1 | C26:0 | 1055.1 | 1037.1 | 642.7 | 50 |
| C26:1 | C27:0 | 1069.0 | 1051.0 | 656.6 | 50 |
| C26:1 | C28:0 | 1083.1 | 1065.1 | 670.7 | 50 |
| C27:0 | C14:0 | 902.9 | 884.9 | 474.5 | 45 |
| C27:0 | C16:0 | 930.9 | 912.9 | 502.5 | 45 |
| C27:0 | C18:0 | 958.9 | 940.9 | 530.5 | 45 |
| C27:0 | C20:0 | 987.0 | 969.0 | 558.6 | 45 |
| C27:0 | C21:0 | 1001.0 | 983.0 | 572.6 | 40 |
| C27:0 | C22:0 | 1015.0 | 997.0 | 586.6 | 45 |
| C27:0 | C23:0 | 1029.0 | 1011.0 | 600.6 | 50 |
| C27:0 | C24:1 | 1041.0 | 1023.0 | 612.6 | 50 |
| C27:0 | C24:0 | 1043.0 | 1025.0 | 614.6 | 45 |
| C27:0 | C25:0 | 1057.0 | 1039.0 | 628.6 | 50 |
| C27:0 | C26:1 | 1069.0 | 1051.0 | 640.7 | 50 |
| C27:0 | C26:0 | 1071.1 | 1053.1 | 642.7 | 50 |
| C27:0 | C27:0 | 1085.0 | 1067.0 | 656.6 | 50 |
| C27:0 | C28:0 | 1099.1 | 1081.1 | 670.7 | 50 |
| C28:0 | C14:0 | 916.9 | 898.9 | 474.5 | 45 |
| C28:0 | C16:0 | 944.9 | 926.9 | 502.5 | 45 |
| C28:0 | C18:0 | 972.9 | 954.9 | 530.5 | 45 |
| C28:0 | C20:0 | 1001.0 | 983.0 | 558.6 | 45 |
| C28:0 | C21:0 | 1015.0 | 997.0 | 572.6 | 40 |
| C28:0 | C22:0 | 1029.0 | 1011.0 | 586.6 | 45 |
| C28:0 | C23:0 | 1043.0 | 1025.0 | 600.6 | 50 |
| C28:0 | C24:1 | 1055.1 | 1037.1 | 612.6 | 50 |
| C28:0 | C24:0 | 1057.0 | 1039.0 | 614.6 | 45 |
| C28:0 | C25:0 | 1071.0 | 1053.0 | 628.6 | 50 |
| C28:0 | C26:1 | 1083.1 | 1065.1 | 640.7 | 50 |
| C28:0 | C26:0 | 1085.1 | 1067.1 | 642.7 | 50 |
| C28:0 | C27:0 | 1099.0 | 1081.0 | 656.6 | 50 |
| C28:0 | C28:0 | 1113.1 | 1095.1 | 670.7 | 50 |

**Supplemental Table S6.** MRM settings for negative ions in the LC/MS/MS analysis of *O*-acyl groups in ω-*O*-acylceramide species.

| ω-*O-*acyl | *N-*acyl | Precursor ions (Q1) | Product ion (Q3) | Collision energy (eV) |
| --- | --- | --- | --- | --- |
|  |  | [M – H]^–^ |  |  |
| *d*_9_-C18:1* | C26:0 | 1012.0 | 290.3 | 25 |
| C16:0 | C28:0 | 1005.0 | 255.3 | 25 |
| C16:0 | C29:0 | 1019.0 | 255.3 | 25 |
| C16:0 | C30:0 | 1033.0 | 255.3 | 30 |
| C16:0 | C31:0 | 1047.0 | 255.3 | 30 |
| C16:0 | C32:0 | 1061.0 | 255.3 | 30 |
| C16:0 | C33:0 | 1075.0 | 255.3 | 30 |
| C16:0 | C34:0 | 1089.0 | 255.3 | 35 |
| C16:0 | C32:1 | 1059.0 | 255.3 | 30 |
| C16:0 | C34:1 | 1087.0 | 255.3 | 30 |
| C16:0 | C36:1 | 1115.0 | 255.3 | 30 |
| C16:0 | C38:1 | 1143.0 | 255.3 | 30 |
| C16:1 | C28:0 | 1003.0 | 253.3 | 25 |
| C16:1 | C29:0 | 1017.0 | 253.3 | 25 |
| C16:1 | C30:0 | 1031.0 | 253.3 | 30 |
| C16:1 | C31:0 | 1045.0 | 253.3 | 30 |
| C16:1 | C32:0 | 1059.0 | 253.3 | 30 |
| C16:1 | C33:0 | 1073.0 | 253.3 | 30 |
| C16:1 | C34:0 | 1087.0 | 253.3 | 35 |
| C16:1 | C32:1 | 1057.0 | 253.3 | 30 |
| C16:1 | C34:1 | 1085.0 | 253.3 | 30 |
| C16:1 | C36:1 | 1113.0 | 253.3 | 30 |
| C16:1 | C38:1 | 1141.0 | 253.3 | 30 |
| C16:1 | C40:1 | 1169.0 | 253.3 | 30 |
| C18:0 | C28:0 | 1033.0 | 283.3 | 25 |
| C18:0 | C29:0 | 1047.0 | 283.3 | 25 |
| C18:0 | C30:0 | 1061.0 | 283.3 | 30 |
| C18:0 | C31:0 | 1075.0 | 283.3 | 30 |
| C18:0 | C32:0 | 1089.0 | 283.3 | 30 |
| C18:0 | C34:0 | 1117.0 | 283.3 | 35 |
| C18:0 | C32:1 | 1087.0 | 283.3 | 30 |
| C18:0 | C34:1 | 1115.0 | 283.3 | 30 |
| C18:0 | C36:1 | 1143.0 | 283.3 | 30 |
| C18:0 | C38:1 | 1171.0 | 283.3 | 30 |
| C18:1 | C28:0 | 1031.0 | 281.3 | 25 |
| C18:1 | C29:0 | 1045.0 | 281.3 | 25 |
| C18:1 | C30:0 | 1059.0 | 281.3 | 30 |
| C18:1 | C31:0 | 1073.0 | 281.3 | 30 |
| C18:1 | C32:0 | 1087.0 | 281.3 | 30 |
| C18:1 | C33:0 | 1101.0 | 281.3 | 30 |
| C18:1 | C34:0 | 1115.0 | 281.3 | 35 |
| C18:1 | C36:0 | 1143.0 | 281.3 | 35 |
| C18:1 | C32:1 | 1085.0 | 281.3 | 30 |
| C18:1 | C34:1 | 1113.0 | 281.3 | 30 |
| C18:1 | C36:1 | 1141.0 | 281.3 | 30 |
| C18:1 | C38:1 | 1169.0 | 281.3 | 30 |
| C18:2 | C26:0 | 1001.0 | 279.3 | 25 |
| C18:2 | C28:0 | 1029.0 | 279.3 | 25 |
| C18:2 | C29:0 | 1043.0 | 279.3 | 25 |
| C18:2 | C30:0 | 1057.0 | 279.3 | 30 |
| C18:2 | C31:0 | 1071.0 | 279.3 | 30 |
| C18:2 | C32:0 | 1085.0 | 279.3 | 30 |
| C18:2 | C33:0 | 1099.0 | 279.3 | 30 |
| C18:2 | C34:0 | 1113.0 | 279.3 | 35 |
| C18:2 | C35:0 | 1127.0 | 279.3 | 35 |
| C18:2 | C36:0 | 1141.0 | 279.3 | 35 |
| C18:2 | C32:1 | 1083.0 | 279.3 | 30 |
| C18:2 | C34:1 | 1111.0 | 279.3 | 30 |
| C18:2 | C36:1 | 1139.0 | 279.3 | 30 |
| C18:2 | C38:1 | 1167.0 | 279.3 | 30 |
| C18:2 | C40:1 | 1195.0 | 279.3 | 30 |
| C18:3 | C26:0 | 999.0 | 277.3 | 25 |
| C18:3 | C28:0 | 1027.0 | 277.3 | 25 |
| C18:3 | C29:0 | 1041.0 | 277.3 | 25 |
| C18:3 | C30:0 | 1055.0 | 277.3 | 30 |
| C18:3 | C31:0 | 1069.0 | 277.3 | 30 |
| C18:3 | C32:0 | 1083.0 | 277.3 | 30 |
| C18:3 | C33:0 | 1097.0 | 277.3 | 30 |
| C18:3 | C34:0 | 1111.0 | 277.3 | 35 |
| C18:3 | C35:0 | 1125.0 | 277.3 | 35 |
| C18:3 | C36:0 | 1139.0 | 277.3 | 35 |
| C18:3 | C32:1 | 1081.0 | 277.3 | 30 |
| C18:3 | C34:1 | 1109.0 | 277.3 | 30 |
| C18:3 | C36:1 | 1137.0 | 277.3 | 30 |
| C18:3 | C38:1 | 1165.0 | 277.3 | 30 |
| C20:4 | C28:0 | 1053.0 | 303.3 | 25 |
| C20:4 | C29:0 | 1067.0 | 303.3 | 25 |
| C20:4 | C30:0 | 1081.0 | 303.3 | 30 |
| C20:4 | C31:0 | 1095.0 | 303.3 | 30 |
| C20:4 | C32:0 | 1109.0 | 303.3 | 30 |
| C20:4 | C33:0 | 1123.0 | 303.3 | 30 |
| C20:4 | C34:0 | 1137.0 | 303.3 | 35 |
| C20:4 | C32:1 | 1107.0 | 303.3 | 30 |
| C20:4 | C34:1 | 1135.0 | 303.3 | 30 |
| C20:4 | C36:1 | 1163.0 | 303.3 | 30 |
| C20:4 | C38:1 | 1191.0 | 303.3 | 30 |

*Internal standard
